# Atomistic mechanism of non-selective cation permeation in cyclic nucleotide-gated CNGA1 ion channel by molecular dynamics simulations

**DOI:** 10.1038/s42003-025-08705-5

**Published:** 2025-08-23

**Authors:** Haoran Liu, Johann Biedermann, Han Sun

**Affiliations:** 1 Research Unit of Structural Chemistry & Computational Biophysics, Leibniz-Forschungsinstitut für Molekulare Pharmakologie, Berlin, Germany; 2 Department of Chemistry, Technische Universität Berlin, Berlin, Germany

**Keywords:** Cyclic nucleotide-gated cation channels, Computational biophysics

## Abstract

Mammalian cyclic nucleotide-gated (CNG) ion channels play a fundamental role in signal transduction within the visual and olfactory sensory cells, converting external stimuli into electrical signals. Here, using large-scale atomistic molecular dynamics (MD) simulations of three different constructs under applied transmembrane voltages, we uncover the atomistic mechanism of monovalent cation permeation in the homotetrameric CNGA1 channel. Owing to the high plasticity and large dimensions of its selectivity filter (SF), monovalent cation binding within the SF of the CNGA1 channel is more dynamic and diffuse compared to that in potassium-selective and hyperpolarization-activated cyclic nucleotide-gated (HCN) channels. K^+^ and Na^+^ permeation in CNGA1 involves hydrated cations passing through the SF with strong occupancy at three regions. In addition, we proposed that the higher Na^+^ occupancy in the SF compare to K^+^ underlies the experimentally observed larger Na^+^ conductance. Our study provides atomistic insights into non-selective cation permeation mechanisms that are not accessible through static structural analysis alone.

## Abstract (web summary)

Atomistic molecular dynamics simulations reveal the mechanism of non-selective cation permeation in the human cyclic nucleotide-gated CNGA1 channel, where hydrated cations traverse a highly dynamic selectivity filter.

## Introduction

Cyclic nucleotide-gated (CNG) ion channels are present in both prokaryotic and eukaryotic genomes. In eukaryotes, they are found in photoreceptors, olfactory sensory neurons, and the central nervous system^1–4^. In response to changes in intracellular concentrations of cAMP or cGMP, which are linked to various biochemical signaling pathways, CNG channels open or close, regulating the flow of cations across the cell membrane^5,6^. Mutations in CNG channels are associated with a variety of channelopathies. For example, mutations in the A1 subunit of human cone CNG channels cause retinitis pigmentosa, characterized by progressive degeneration of rod and cone receptors, leading to partial or full blindness^7,8^.

CNG channels are non-selective cation channels that do not discriminate among monovalent cations and even allow the permeation of divalent cations such as Ca^2+^. However, the opening time of the channel is largely influenced by the presence of different cation types, with longer opening time for K^+^ than Na^+^^9,10^. At the single-channel level, Na^+^ revealed approximately 1.8 times larger unitary conductance compared to K^+^. Furthermore, both Na^+^ and K^+^ showed a wide conductance distribution in the open state, which was hypothesized to be caused by pore fluctuation^10^. Although CNG channels possess a voltage-sensing domain (VSD), they display little voltage sensitivity in their macroscopic currents, which is in strong contrast to their structural homologs, hyperpolarization-activated cyclic nucleotide-gated (HCN) channels^11–14^.

**Fig. 1:**
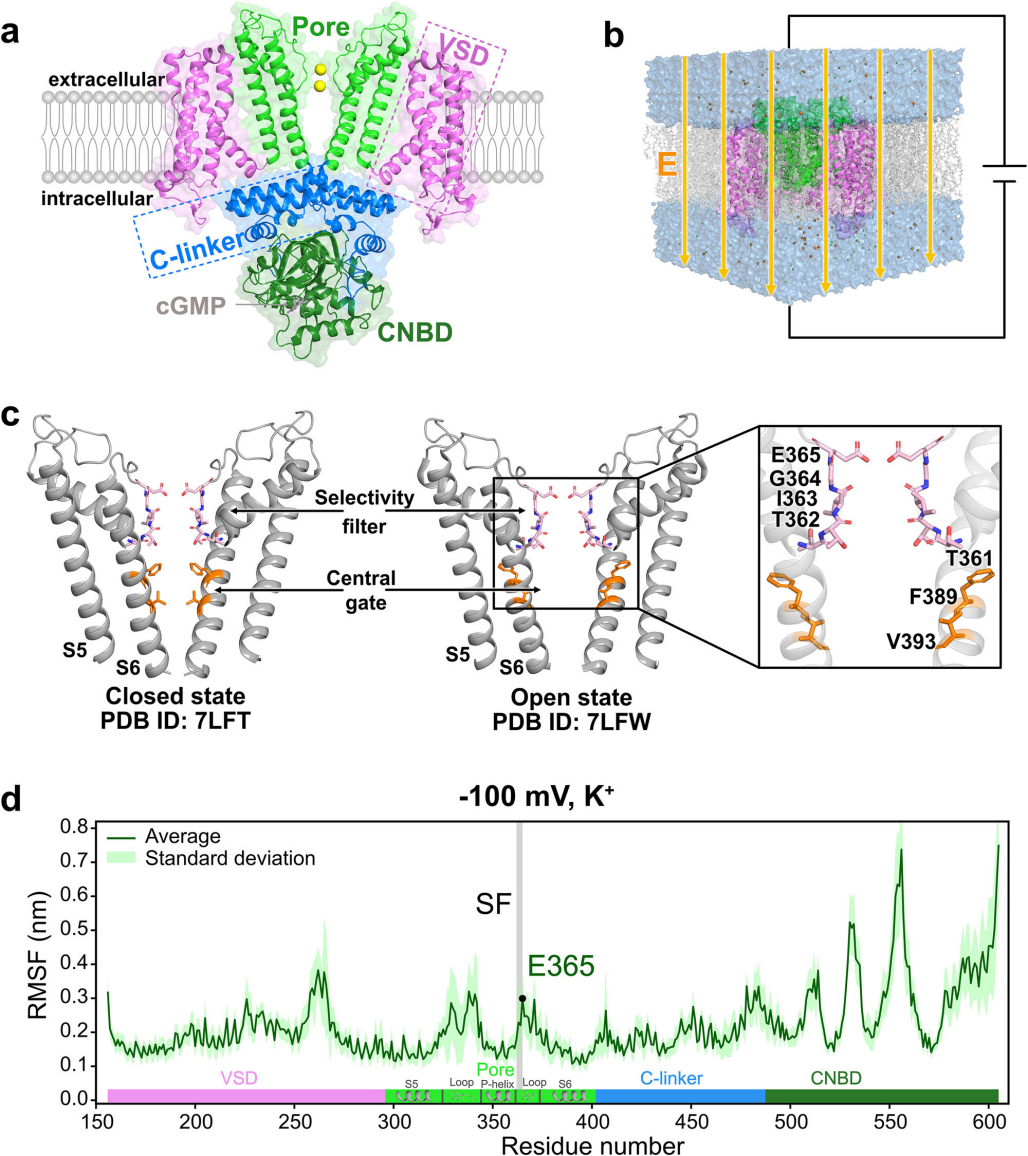
Overview of CNGA1 channel structure and MD simulation setup. **a** Structure of the cGMP-bound CNGA1 channel (PDB ID: 7LFW)^27^ within the membrane. The channel is a tetramer composed of four subunits, including the pore domain (green), voltage-sensor domain (VSD, pink), C-linker (blue), and cyclic nucleotide-binding domain (CNBD, deep green), with cGMP depicted as gray sticks. For clarity, only two diagonally opposed subunits are shown. **b** The transmembrane potential during the MD simulation is established by applying an external electric field across the simulation box^31^. **c** Structural details of the pore domain in both the open (PDB ID: 7LFW) and closed (PDB ID: 7LFT) conformations^27^. Key residues in the selectivity filter (pink) and central gate residues (orange) are highlighted and labeled. **d** Root-mean-square-fluctuation (RMSF) of the MD simulations employed the whole CNGA1 channel with K^+^ under -100 mV. The green line represents the average RMSF value, while the light green shading indicates the standard deviation from five independent simulation runs. The gray-highlighted region marks the SF including E365 at the extracellular tip.

Structurally, CNG channels exist as both homo- and heterotetramers, with native human CNG channels forming heteromers consisting of homologous alpha (A) and beta (B) subunits^15–17^. In rod photoreceptor, the native CNG channel consists of three A1 subunits and one B1 subunit, while the cone CNG channel is formed by A3 and B3 subunits in a stoichiometry of either 3:1 or 2:2. In olfactory sensory neurons, the native CNG comprises two A2 subunits, one A4 subunit, and one B1b subunit^18–23^. Each A and B subunit includes a pore domain, a voltage-sensing domain (VSD), a C-terminal linker (C-linker), and a cyclic nucleotide-binding domain (CNBD) (Fig. 1a). Four residues in the pore domain, T362, I363, G364, and E365, form the selectivity filter (SF), which allows only cations but not anions to pass through the channel (Fig. 1c). The first atomistic structures of the apo closed and cGMP-bound open states of CNG channels were resolved for TAX-4, a homomeric CNG channel present in *C.elegans*^24,25^. Later, structures of human homomeric CNGA1, heteromeric CNGA1/B1 rod channel, and the cone CNGA3/B3 channel in the ligand-bound open and apo closed states were resolved^22,26–28^. These structures revealed an unprecedented but conserved gating mechanism among different CNG channels governed by a single central cavity gate involving two hydrophobic residues, for example F389 and V393 in case of CNGA1 (Fig. 1c). The SF conformations do not differ between the open and closed states of these CNG channels, resolving a long-standing debate about whether the SF functions as a gate, similar to some potassium channels^29,30^.

Based on the first open and closed structures of human CNGA1 channels, we investigated here the atomistic mechanisms of monovalent cation permeation in CNGA1 by performing the atomistic molecular dynamics (MD) simulations under transmembrane voltages^31^ (Fig. 1b). In recent years, MD simulations have become an indispensable theoretical approach, bridging high-resolution structural analysis and electrophysiological characterization of ion channels. These computational studies together with the experimental validation enabled a detailed understanding of the atomistic mechanisms of ion permeation, gating, pharmacological modulation, and post-transcriptional regulation of various ion channels^32–34^. In this study, we demonstrated that the non-selective permeation of Na^+^ and K^+^ in the CNGA1 channel involves three primary ion-binding regions located within and around the SF region, where partial dehydration of the cations’ first water shell occurs in the narrow pore. Based on our MD simulations, we attribute the higher single-channel conductance of Na^+^ compared to K^+^ to increased ion occupancy within the SF and pore, which facilitates a more efficient knock-on mechanism for cation permeation.

## Results

### Main ion binding profiles during ion permeation

We performed a large number of atomistic MD simulations starting from the open (PDB ID: 7LFW) and closed states (PDB ID: 7LFT) of the CNGA1 channel^27^, using both K^+^ and Na^+^ under a range of positive and negative voltages (Supplementary Table S1). In this study, we used three different constructs (Fig. 1a): (i) the pore domain only, a commonly adopted strategy in many previous computational ion channel studies to reduce computational cost ^35–37^; (ii) the complete transmembrane domain, which includes the pore domain and the VSD; and (iii) the full-length channel construct. The CNGA1 channel structures were embedded within a palmitoyloleoyl phosphatidylcholine (POPC) lipid bilayer and subjected to an external electric potential to establish transmembrane voltages^31^. The simulations were mainly performed using the CHARMM36m force field^38^, with additional simulations conducted using the AMBER99SB^39^ and AMBER19SB^40^ force fields for validation purposes (Supplementary Table S1, S2).

**Fig. 2:**
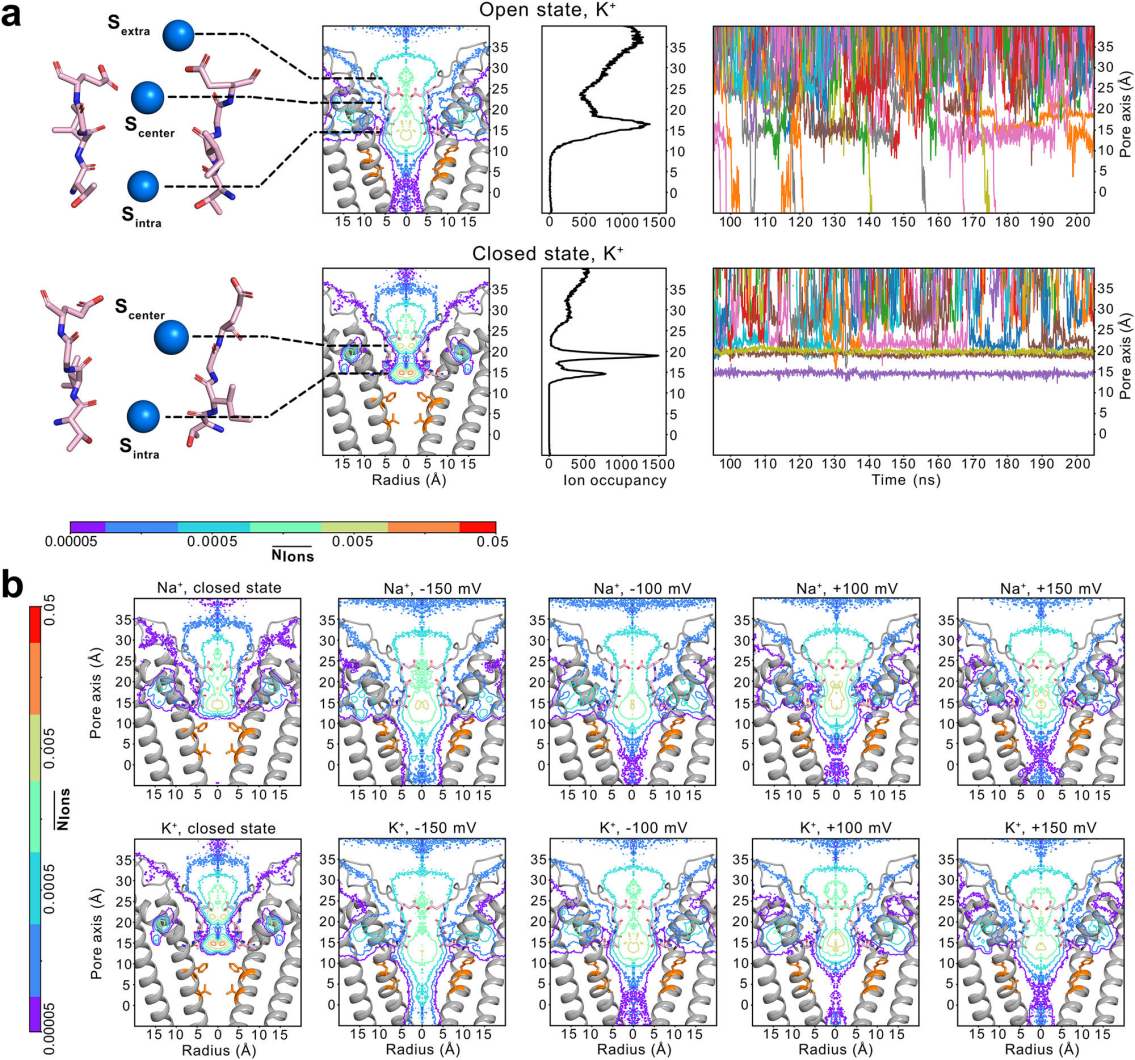
Ion binding profiles of K^+^ and Na^+^ within the ion conduction pathway derived from atomistic MD simulations. **a** (1st column) Major cation-binding regions within the SF; (2nd column) 2-dimensional K^+^ ion occupancy maps along the pore; (3rd column) 1-dimensional K^+^ ion occupancy profiles along the pore; and (4th column) representative traces showing K^+^ trajectory through the pore of the CNGA1 channel in the (top) open state and (bottom) closed state CNGA1 channel. **b** Comparison of 2-dimensional ion occupancy in the CNGA1 channel pore in closed (1st column) and open states (2–4 column), under varying voltage conditions with Na^+^ and K^+^. The ion occupancy in number of ions per 0.001 Å^3^ per 50 ps was normalized according to the volume change along the radius. Ion binding profiles were derived from the aggregated simulation replicas of the pore-domain construct of CNGA1 using the CHARMM36m force field. The corresponding profiles from the simulations of the pore+VSD construct are shown in Supplementary Fig. S2.

In the simulations, we observed a large number of K^+^ and Na^+^ permeation events in the open state of the CNGA1 channel during the simulated timescale, while no permeation occurred in the closed state simulations on the same duration (Fig. 2a; exemplary ion conduction events of K^+^ and Na^+^ in CNGA1 are shown in Supplementary Movie S1 and S2, respectively). Along the ion conduction pathway in the open state, cation occupancy was primarily concentrated within the SF region, with very low ion occupancy in the central cavity region, likely due to its highly hydrophobic nature. Interestingly, the selectivity filter exhibited high flexibility in the simulations of all three constructs, with RMSF value reaching up to 0.3 nm, which is comparable to those of some free loop regions (Fig. 1d, Supplementary Fig. S1). This pronounced flexibility of the SF resulted in highly dynamic and diffusive K^+^ and Na^+^ binding, particularly in the open-state simulations where ion conduction occurred (Fig. 2a, Supplementary Movie S1 and S2). This behavior contrasts with the more tightly coordinated K^+^ binding observed during conduction observed in K^+^-selective and hyperpolarization-activated cyclic nucleotide-gated (HCN) channels studied using similar computational approaches^41–45^.

We conducted a more detailed examination of the ion-binding profiles in the SF of the CNGA1 channel, based on MD simulations, and identified three major ion-binding regions: (i) at the lower end of the SF, coordinated by the backbone carbonyls and/or side chains of T362 residues, denoted as S_intra_, which closely corresponds to *site 2* in the previous cryo-EM analysis of CNGA1 channel^27^; (ii) in the central part of the SF, surrounded by the backbone carbonyls of I363 and partially by the side chains of E365, denoted as S_central_, which corresponds to *site 1* in the cryo-EM analysis^27^; and (iii) at the extracellular entrance of the filter, coordinated by the side chains of the negatively charged E365, denoted as S_extra_ (Fig. 2a). The electron density for this binding site was very weak in the cryo-EM map and was not further analyzed^27^, which is rather unexpected given the acidic nature of E365. Interestingly, a prominent cation binding site at the corresponding position was observed in the closed-state structure of the TAX-4 CNG channel^25^. From the MD simulations, we found that the binding at the extracellular entrance is pronounced for both K^+^ and Na^+^ under both negative and positive transmembrane potentials (Fig. 2b and Supplementary Fig. S2). However, this binding site is even more dynamic compared to the binding regions within the SF due to the high mobility of the E365 side chain (Fig. 1d, Supplementary Fig. S1). Therefore, we hypothesize that the absence of this binding site in the cryo-EM analysis of CNGA1 is likely due to its dynamic nature. Notably, because of the highly dynamic and diffusive binding of the ions within the filter, it is difficult to discern clear differences in ion occupancy between K^+^ and Na^+^. In general, both cations occupy very similar regions within the SF during conduction (Fig. 2b).

In addition, our simulations revealed a strong Na^+^ and K^+^ binding site located behind the SF, enable by the backbone of W355 and side-chains of T359 and D381 (Supplementary Fig. S3, Supplementary Movie S1). Notably, in simulations conducted at +100 mV, approximately 8% of K^+^ ions entered the SF via this side-pocket binding site rather than the canonical on-axis pathway (Supplementary Table S3). Although this binding site is prominent in our simulations, it was not resolved in the cryo-EM structures of the CNGA1 channel. Whether this site contributes to the stability of the filter or plays a direct role in ion conduction remains unclear. Future mutagenesis studies, both in simulations and experiments, are needed to clarify its functional relevance.

### Hydration state of ions during permeation

**Fig. 3:**
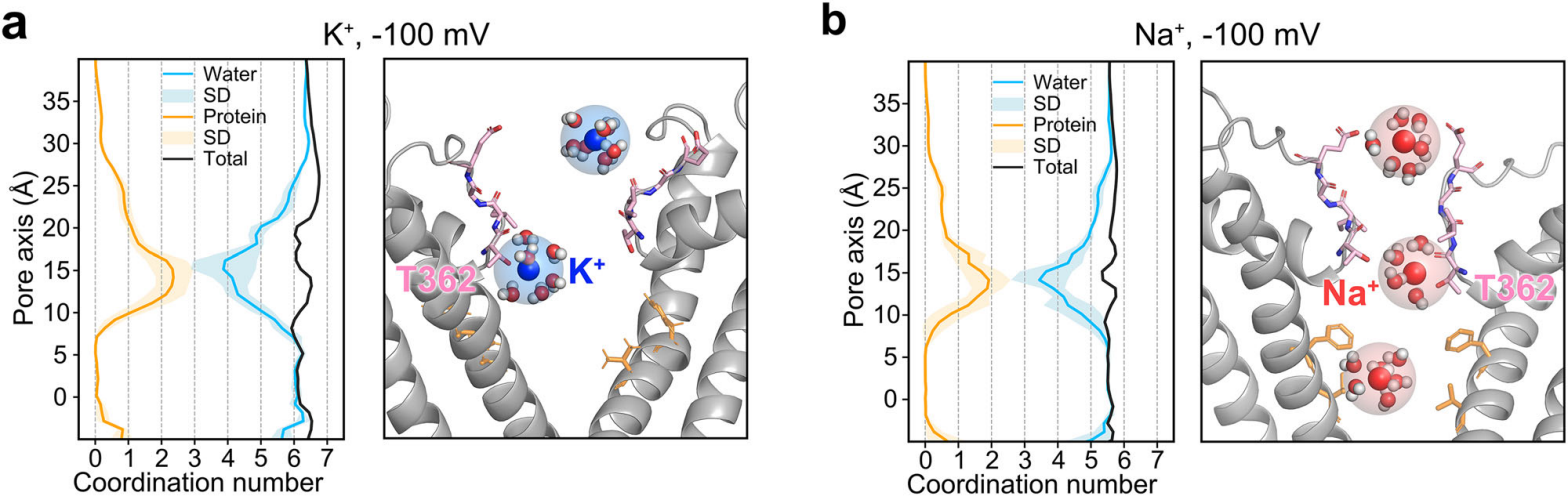
Hydration profiles of Na^+^ and K^+^ during their permeation in CNGA1 channel. **a**, **b** (left) Ion hydration profiles of K^+^ and Na^+^, respectively. The orange line represents the average protein oxygens within the first hydration shell of the cation and the light orange shading indicates the standard deviation from five independent simulation runs. The blue line represents the average water oxygens within the first hydration shell and the light blue shading indicates the standard deviation from five independent simulation runs. The black line represents the total number of oxygens within the first hydration shell of the ion. The snapshot (right) shows the representative snapshots selected from K^+^ (blue) and Na^+^ (red) simulations, respectively. The first hydration shell is represented as a transparent sphere. The simulations were performed with the pore-only construct of CNGA1 using the CHARMM36m force field.

Next, we characterized the hydration state of K^+^ and Na^+^ ions during their permeation. Due to the considerably wider dimension of the SF of CNG channels compared to that of K^+^ channels, it was hypothesized that the cations pass through the CNG filter in a partially hydrated form^27,46^, which is in strong contrast to K^+^ channels and also to NaK, a non-selective cation channel considered to be a bacterial homolog of CNG and HCN channels^47–49^. The analysis of our MD simulations aligned well with this hypothesis and showed that both K^+^ and Na^+^ become partially dehydrated in the filter of the CNGA1 channel, exhibiting highly comparable hydration profiles for K^+^ and Na^+^ (Fig. 3). At their major binding sites in the SF, cations lose a maximum of three water molecules, which are replaced by coordination with two to three protein oxygens. At the central cavity gate, cations always retain their full hydration state during permeation.

Previous studies have suggested that the solvation difference between K^+^ and Na^+^ underlies the high K^+^ selectivity in K^+^-selective channels. Our study supports this conclusion, demonstrating that partially hydrated K^+^ and Na^+^ can permeate through the SF of the CNG channel, thereby providing a mechanistic basis for its non-selective permeability to monovalent cations.

### Comparison of simulated and experimental conductance

**Fig. 4:**
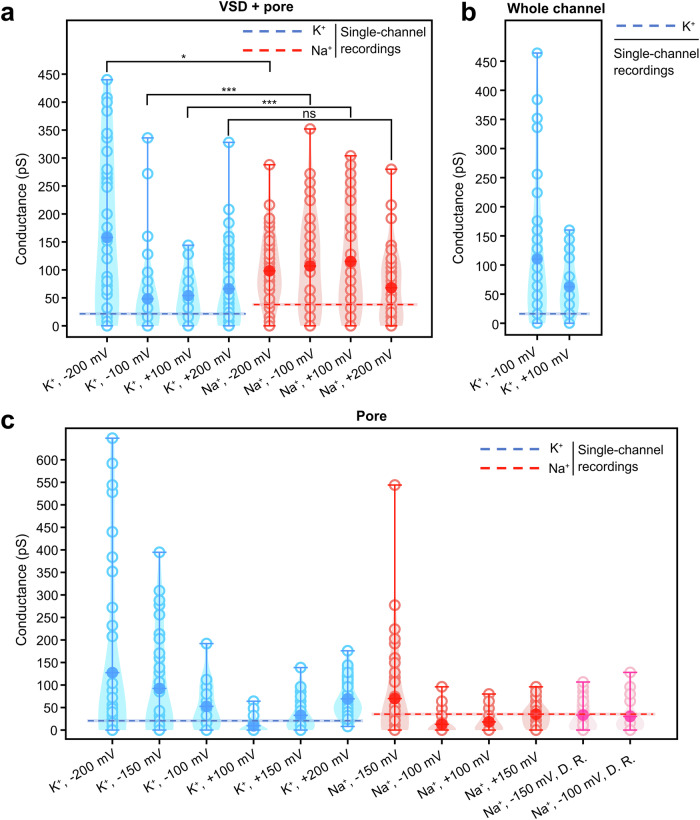
Comparison of conductance across different simulation setups. **a** Conductance derived from simulations of the pore domain and the voltage-sensor domain (VSD) of the CNGA1 channel with K^+^ (blue) and Na^+^ (red) under various transmembrane voltages. **b** Conductance derived from simulations of the whole CNGA1 channel with K^+^ under –100 mV and +100 mV. **c** Conductance derived from simulations of pore domain only of the CNGA1 channel with K^+^ (blue) and Na^+^ (red, pink) under different transmembrane voltages. “D. R.” represents simulations with distance restraints on gate residues F389, based on distances of the open state in the cryo-EM structures^27^. Open circle represents the simulated conductance from each 100 ns segment, while the closed circle shows the averaged value from all data points. Conductance from experimental single-channel recordings for K^+^ (blue, 20.0 ± 1.3 pS, +100 mV) and Na^+^ (red, 36.0 ± 1.7 pS, +100 mV) from a previous study^10^ are shown as dashed lines, with error margins represented by shaded areas. A statistical *t* test was performed from 45 segments for each simulation setup (each 1 µs trajectory was divided into 10 segments and the first 100 ns segment was considered as equilibration phases and discarded), and *p* values were calculated to assess the significance of the difference between the simulated K^+^ and Na^+^ conductance in the pore+VSD construct. Sample size for each simulation setup: 45. [The exact *p* values: -200 mV: 0.0103; -100 mV: 0.0006; +100 mV: 0.0001; +200 mV: 0.8919. Not significant (ns): *p* > 0.05; significant (*): *p* ≤ 0.05; very significant (**): *p* ≤ 0.01; highly significant (***): *p* ≤ 0.001]. The simulated current-voltage relationships of the pore domain alone and pore+VSD constructs are shown in Supplementary Fig. S4.

Since a high number of K^+^ and Na^+^ permeation events were observed in each simulation run (up to 400), we divided each 1 µs trajectory into 10 segments and calculated the conductance from each 100 ns segment to obtain better statistical estimates of the simulated conductance (Fig. 4, Supplementary Fig. S4, open circle represents the simulated conductance from each 100 ns segment, while the closed circle shows the averaged value from all data points). When comparing the simulated conductance of the pore-only simulations, K^+^ conductance at physiologically relevant transmembrane voltages (–100 mV and 100 mV) falls within the range of the experimentally measured single-channel conductance for K^+^, which is 20.0 ± 1.3 pS at +100 mV under saturating cGMP concentration^10^. In contrast, the simulated Na^+^ is considerably lower than the experimental conductance of 36.0 ± 1.7 pS for Na^+^. Simulated K^+^ and Na^+^ conductance at –200 mV and –150 mV revealed irregular high conductance with large fluctuations across different simulation runs, which may be attributed to pronounced pore fluctuation induced by the unphysiological transmembrane voltages. When distance restraints were applied to the gate residues during the simulations at -150 mV, this irregular high conductance was markedly reduced (Fig. 4c).

We also compared the simulated conductance of the pore+VSD and whole-channel constructs with experimental values. Due to the high computational cost of simulating the full channel, only K^+^ permeation was simulated under +100 mV and –100 mV. The pore+VSD simulations at physiologically relevant voltages ( + 100 mV and –100 mV) exhibited approximately twice as high as the experimental values for both K^+^ and Na^+^. In contrast, the whole-channel constructs showed conductance levels three to five times higher than those observed experimentally.

All simulations discussed above were performed with the E365 residues of all four subunits in their deprotonated state. Since Morill and MacKinnon previously proposed that E365 functions as a titratable residue along the ion permeation way^50,51^, we additionally performed simulations in which either two diagonal E365 residues or all four were protonated. When two diagonal E365 residues were protonated, the average conductance increased by 1.5- to 2-fold compared to the fully deprotonated form. Protonation of all four E365 residues resulted in a 2- to 3-fold increase in average conductance (Supplementary Fig. S5). These results suggest that protonation of E365 lowers the energy barrier for cation permeation, likely by modulating electrostatic interactions at the external site of the SF.

### Ion conductance influenced by fluctuation of central cavity gate

**Fig. 5:**
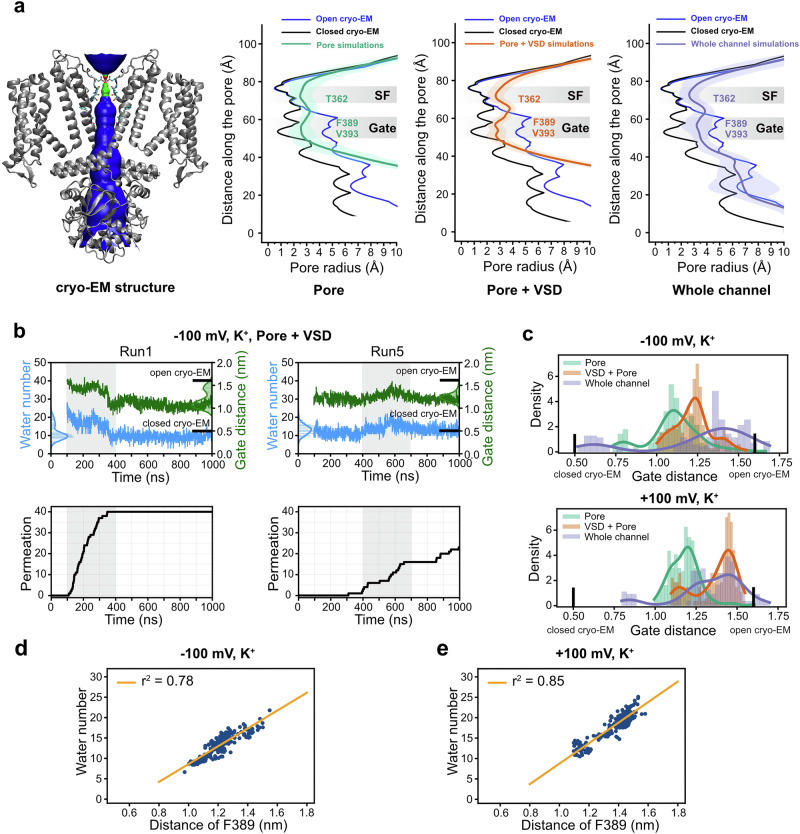
Central cavity gate fluctuation. **a** Pore radius profiles along the central axis of cryo-EM structure and during simulations of different constructs using HOLE program version 2.0^52,53^. The deep-colored line represents the average value and the light-colored shading represents the standard deviation of 10,000 snapshots from five independent simulation runs. **b** Two representative simulation runs illustrating the relationship between the number of water molecules in the gate region, the distance between the two opposing gate residues (F389), and the number of ion permeation events. The distribution of the number of water molecules in the gate region and the distance between the two opposing gate residues (F389) are shown along the y-axis. **c** The distribution of the minimum distance between opposing F389 across different simulation setups. The relationship between the average number of water molecules in the gate region per 20 ns interval and the average distance between the two opposing gate residues (F389) during the same time interval, in total 225 data points from five independent simulation runs (the first 100 ns segment was considered as equilibration phases and discarded). K^+^ permeation simulations were performed at **d** –100 mV and **e** + 100 mV, respectively. The simulations were performed with the pore domain+VSD.

As discussed above, we observed significant fluctuations in ion conductance across different simulation runs (Fig. 4a). Upon examining individual trajectories, we noted that in some simulations, ion permeation ceased after a certain period, while in others the channel remained conductive throughout the entire 900 ns simulation (Fig. 5b, Supplementary Fig. S6). To assess whether gate closure occurred during the simulations, we analyzed pore profiles and gate distances from the three different simulation setups using HOLE program version 2.0^52,53^ (Fig. 5a). These analyses revealed marked deviations of the pore profiles compared to the original cryo-EM structures. Specifically, the SF region became significantly wider, while the gate region becomes narrowed, particularly in the simulations of the pore domain only and the pore+VSD constructs. In contrast, the gate region remained comparably wider in the simulations in the full-channel simulations, though still considerably narrower than in the cryo-EM structure.

This finding is further supported by analysis of the gate distance distributions obtained from simulations of the three different constructs. It becomes evident that under -100 mV the highest gate distances were observed in the full channel simulations, although with a broad distribution (Fig. 5c). In contrast, the pore domain construct showed the lowest gate distances, while the VSD+pore construct exhibited intermediate values. Notably, the simulated conductance values for the pore domain alone and the pore+VSD construct—which both showed more pronounced gate tightening—are more consistent with previously reported experimental single-channel conductance values. In comparison, the simulated conductance of the full channel is 3–5 times higher than the experimental value.

Although the gate distances became narrower in the simulations, the channel did not return to a closed state for the majority of the simulation time. Even in simulations where ion permeation ceased, the minimum gate distance remained above 10 Å, which should, in principle, still permit the passage of hydrated K^+^ ions through the pore. However, we observed that pore narrowing often correlated with a reduction in the number of water molecules within the pore (Fig. 5b, Supplementary Figs. S7, S8). Especially, when we correlated the number of water molecules and the gate distances from the simulations, we obtained a high correlation with r^2^ up to 0.85 (Fig. 5e, Supplementary Figs. S9, S10). We also attempted to correlate gate distance and water occupancy in the gate region with ion conductance (Supplementary Figs. S9, S10); however, the result remained inconclusive. Further studies are needed to determine whether the level of pore dehydration is the directly linked to conductance.

### Higher Na^+^ conductance in comparison to K^+^

When comparing the conductance from simulations that include both the pore domain and the VSD, Na^+^ exhibited a notably higher conductance than K^+^. According to the *t* test, this difference is statistically highly significant (*p* ≤ 0.001) for simulations conducted at voltages ( + 100 mV and –100 mV) close to physiological conditions. Additionally, we observed larger fluctuations in conductance across different simulation runs for Na^+^ compared to K^+^ (Fig. 4a). Although the simulated Na^+^ conductance is significantly higher than the experimental values, it qualitatively matches the experimental trend, which also shows higher Na^+^ conductance than for K^+^.

**Fig. 6:**
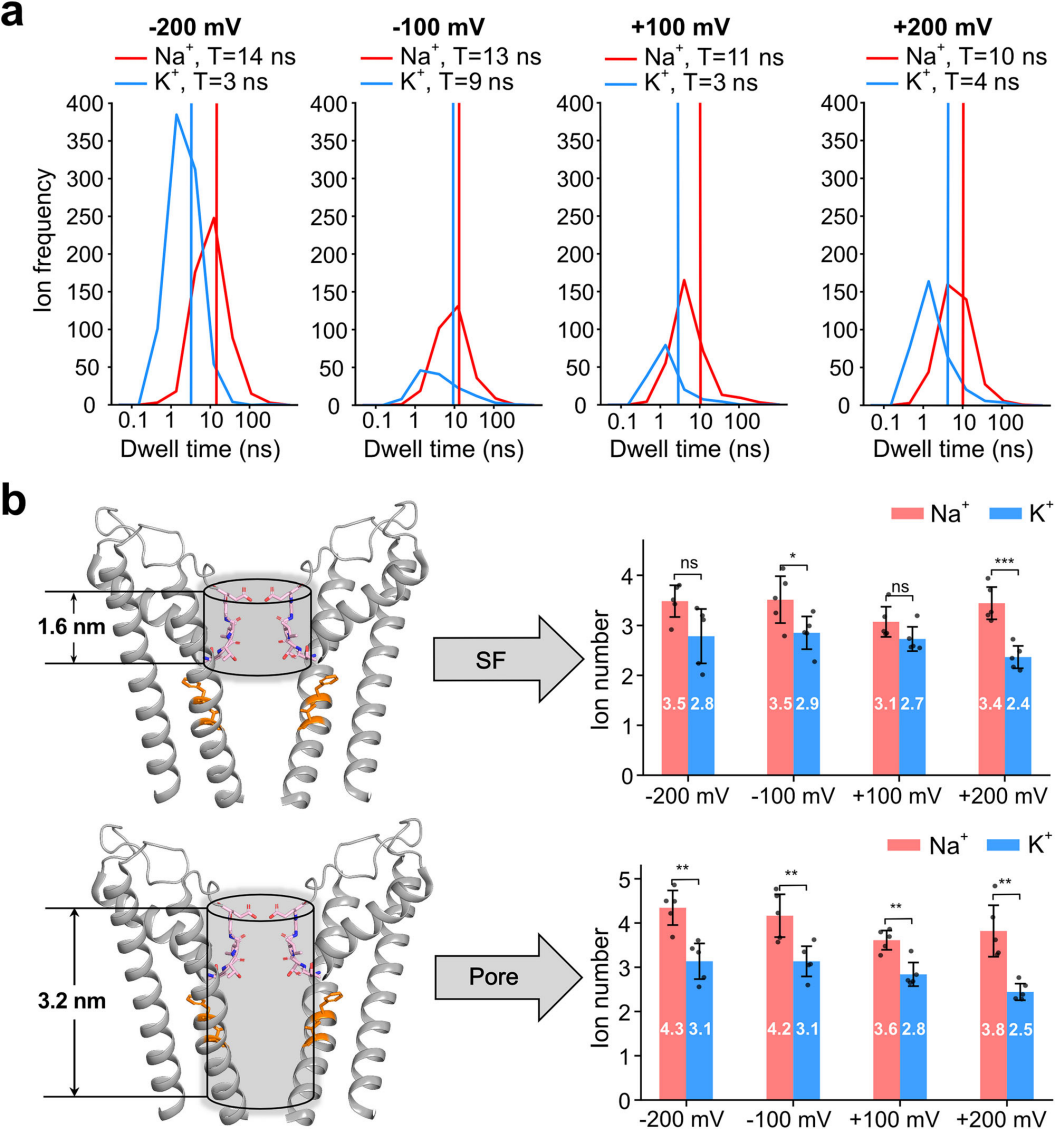
Ion dwell time and density in the pore. **a** Distribution of dwell times for Na^+^ (red) and K^+^ (blue) ions traversing the pore at various transmembrane voltages, with vertical lines indicating the means of the respective dwell times. **b** Average number of Na^+^ (red) and K^+^ (blue) in the SF and pore at different voltages. Simulations were performed with the pore domain and VSD. A statistical *t* test was performed from five independent simulation runs, and *p* values were calculated to assess the significance of the difference of K^+^ and Na^+^ ion number. [The exact *p* values: SF, –200 mV: 0.0564; –100 mV: 0.0487; +100 mV: 0.1140; +200 mV: 0.0006. Pore, –200 mV: 0.0026; –100 mV: 0.0083; +100 mV: 0.0020; +200 mV: 0.0020. Not significant (ns): *p* > 0.05; significant (*): *p* ≤ 0.05; very significant (**): *p* ≤ 0.01; highly significant (***): *p* ≤ 0.001]. All the error bars represent the standard deviation of the mean.

We next investigated why Na^+^ exhibits a larger conductance than K^+^ in the MD simulations of the pore domain and the VSD. It has been previously proposed that Na^+^ has a shorter dwell time in the pore compared to K^+^^10^. To test this hypothesis, we calculated the dwell times of K^+^ and Na^+^ as they traversed the pore in ion permeation simulations. Contrary to the initial proposal, our simulations consistently showed that Na^+^ had a longer dwell time in the pore than K^+^ across all transmembrane voltages examined (Fig. 6a). To further understand this discrepancy, we quantified the average number of ions present in the SF and the entire pore region for both Na^+^ and K^+^. The results revealed that multiple cations simultaneously occupy the filter, as also confirmed by visual inspection (Supplementary Movie S1 and S2), consistent with a multi-ion knock-on mechanism. Moreover, Na^+^ exhibited a higher average occupancy than K^+^ in both the SF and the pore region (Fig. 6b). This difference is particularly pronounced and statistically significant in the pore regions (*p* ≤ 0.01, *t* test). Based on these MD results, we propose that the higher single-channel conductance of Na^+^ arises from its increased density in the pore, which enhances ion–ion interactions and facilitates a more efficient knock-on mechanism during permeation.

## Discussion

In this study, we conducted microsecond atomistic MD simulations to investigate the permeation of monovalent cations in the human CNGA1 channel. To the best of our knowledge, this is the first MD study to address ion permeation and conductance in native CNG channels. Leveraging the recent high-resolution cryo-EM structures of the open and closed states of the CNGA1 channel^27^, our study provides key atomistic mechanistic insights into the dynamics of ion permeation that are not accessible through the analysis of static cryo-EM structures.

By analyzing the ion binding profiles within the SF, we identified ion-binding regions comparable with previous cryo-EM analyses, but also observed an additional pronounced cation-binding region on the extracellular side of the SF. This site appears to be stabilized by the negatively charged and mobile side chain of E365, but was not resolved in the cryo-EM data. We hypothesize that this may be due to the high conformational dynamics of this region or the protonation of E365 under the physiological conditions. The overall dimensions of the SF in the CNGA1 channel are significantly wider than those of typical K^+^-selective and HCN channels. Our simulations also revealed high conformational flexibility of the SF on the nanosecond timescale. As a result, cation binding with the SF region is generally more dynamic and diffusive than in K^+^ and HCN channels ^41,42^. Consequently, we conclude that the SF of CNGA1 does not present a high energy barrier for monovalent cation conduction.

When analyzing the monovalent cation conduction pathway, we identified an additional strong binding site for both K^+^ and Na^+^ located behind the SF. However, across different simulation setups, only a small percentage of ions entered the SF via this off-axis binding site (Supplementary Table S3). At present, we are unable to confirm the functional relevance of this site, and more detailed computational investigations and experimental mutagenesis are needed to clarify its role. Nevertheless, a similar off-axis permeation pathway was also predicted in MD simulations of the cation non-selective NaK channel^49^, where a side-entry ion conduction route for Na^+^ originally predicted from MD simulations was later confirmed by high-resolution X-ray structural analysis^54^. In addition, our recent MD study of the AMPA GluA2 receptor, investigating the effects of Q/R editing within the SF, also predicted a comparable off-axis pathway coexisting with the canonical cation conduction route^55^.

Our MD simulations using three different constructs consistently revealed that partially hydrated Na^+^ and K^+^ pass through the SF. This contrasts with K^+^-selective channels, where near complete dehydration of K^+^ has been proposed as a key mechanism underlying their high K^+^ selectivity. A previous study using a CNG pore mimic based on the NaK scaffold suggests that the pore flexibility underlies the poor selectivity of the CNG channel^56^. While our study on the native CNG channel also demonstrated pronounced SF flexibility, we propose that the ability of both Na^+^ and K^+^ to permeate through the SF without significant differences in their hydration states is a key contributor to the lack of selectivity among monovalent cations. Furthermore, it is noteworthy that in some other non-selective cation channels, such as the NaK channel^49^ and the lysosomal two-pore channel 2 (TPC2)^57^, additional conformational changes in the SF are required to accommodate both K^+^ and Na^+^ in their hydrated forms as they traverse the narrowest region of the pore.

In contrast to many other computational investigations of ion channels that employed substantially larger transmembrane voltages (300–600 mV) to accelerate the ion permeation process, we used here a range of transmembrane voltages close to those in experimental electrophysiology studies ( ±100–±200 mV). For comparison, we also performed ion permeation simulations of CNGA1 with the AMBER99SB and AMBER19SB force fields. Overall, we observed lower conductance in the AMBER simulations, particularly an unrealistically low Na^+^ conductance (Supplementary Table S1), which was also seen in our previous simulations with the AMPA GluA2 receptor ion channel^37^.

In simulations of the construct including the VSD and pore, the simulated Na^+^ conductance was notably higher than that of K^+^, which also qualitatively aligns with experimental observations. From the MD simulations, we observed 2–4 K^+^ and Na^+^ simultaneously occupying the SF, with higher Na^+^ density in the SF and pore. However, due to very pronounced flexibility of the SF, it is difficult to clearly discern which residues are responsible for the differences in Na^+^ and K^+^ occupancy. Interestingly, CNGA1 channel share the same tetrameric EEEE rings in their SF as bacterial voltage-gated sodium (Na_v_) channels. It has been suggested that the strong charge-donating ability of the Glu carboxylates enables Na^+^ to outcompete the weaker electron-acceptor K^+^, thereby contributing to the higher Na^+^ selectivity in Na_v_ channel^58^. Based on this, we speculate that the EEEE outer ring also facilitates the recruitment of more Na^+^ ions into the SF of CNGA1 channel compared to K^+^. Consequently, we propose that the higher Na^+^ density in the pore facilitates multi-cation knock-on mechanism within the narrow ion conduction pathway of CNGA1, contributing as the primary mechanism underlying the higher single-channel conductance of Na^+^ than K^+^. Notably, a recent study on Ca^2+^ permeation in Ca_v_1 channel also highlighted the importance of a multi-ion knock-on mechanism within the SF for efficient ion permeation, as supported by potential of mean force calculations using metadynamics simulations^59^. A comparable mechanism has been proposed, based on Brownian dynamics simulations, to explain the rectification behavior of cation channels^60^. In line with this, our recent computational and electrophysiology investigation of the different mHCN subtypes demonstrated that a higher cation density in the outer channel vestibule promotes an efficient knock-on mechanism, contributing to the elevated ion conductance in the mHCN2 channel compared to other mHCN subtypes^45^.

Our simulations using three different constructs consistently revealed pronounced pore dynamics. Even in the simulations of the full channel, we observed a widening of the SF accompanied by tightening of the gate, an effect that was even more pronounced in the simulations of the pore-only and pore+VSD constructs. It should be noted that this gate tightening did not fully close the gate to prevent cation passage. While we observed a strong correlation between gate distance and water occupancy in the gate region, it remains unclear whether partial dehydration is directly responsible for the observed slowdown in ion conduction. Further studies are needed to understand the variation in ion conductance observed in MD simulations, which may be related to the high single-channel noise characteristic of CNG channels.

Another surprising finding from our simulations is the relatively high conductance compared to the experimental single-channel data. Among all constructs, only the pore domain simulations, which showed significant gate tightening, produced conductance values that approximated experimental levels. In contrast, simulations of the full-channel, where gate tightening was much less pronounced, resulted in conductance values 3–5 times higher than the experimental measurements. This finding stands in stark contrast to many prior simulation studies of K^+^-selective channels using classical additive force fields, which typically yield K^+^ conductance values an order of magnitude lower than experimental measurements. Notably, only a recent study employing charge scaling was able to quantitatively reproduce the experimental conductance^61^. Compared to the K^+^-selective channels, CNGA1 channel possesses a considerably wider SF and exhibits greater structural flexibility, with water molecules mediating the interactions between the ions and the filter. These features may make it inherently more difficult to quantitatively reproduce the experimental single-channel conductance. Nevertheless, future study applying charge scaling strategies or employing polarizable force field parameters are warranted to further investigate this process.

In conclusion, large-scale atomistic MD simulations of ion permeation in the CNGA1 channel confirmed that the open state of CNGA1, as resolved by cryo-EM, represents the conductive state. We identified the SF as the principal barrier for monovalent cations, with permeation proceeding via a multi-ion knock-on mechanism. The simulations revealed that both K^+^ and Na^+^ traverse the pore primarily by binding in the SF region in a partially dehydrated form, providing atomistic insights into cation non-selectivity. Moreover, we observed a higher Na^+^ density in the SF compared to K^+^, which likely facilitates more efficient multi-ion knock-on events and may underlie the higher single-channel conductance observed for Na^+^.

## Methods

### Atomistic molecular dynamics simulation

In this study, MD simulations started from the high-resolution cryo-EM structure of the homomeric CNGA1 channel in the open state (PDB ID: 7LFW) and the closed state (PDB ID: 7LFT)^27^. The CNGA1 channel was simulated using three different structural models: the full-length channel construct (Fig. 4b), the full transmembrane domain, encompassing both the pore domain and the voltage-sensing domain (VSD) (Fig. 4a); an isolated pore domain model (Fig. 4c), a strategy frequently employed in MD simulations of ion channels to mitigate computational complexity^35–37^. To prevent any artifacts originating from charges at the N- and C-termini, the structures were neutralized by adding acetyl and N-methyl caps using PyMOL^62^ Builder option.

The simulations were mainly performed using the CHARMM36m force field^38^, while several simulations were conducted with the AMBER99SB^39^ and AMBER19SB^40^ force fields for validation purposes (Supplementary Table S1). The CNGA1 channel structures were embedded within a palmitoyloleoyl phosphatidylcholine (POPC) lipid bilayer. In the AMBER99SB simulation setup, we inserted the CNGA1 channel into the POPC lipid bilayer with the GROMACS internal embedding function. Improved ion parameters^63^ and lipid parameters^64^ were employed in these simulations. The insertion of CNGA1 channel into the lipid bilayer was carried out in CHARMM-GUI^65^ for AMBER19SB and CHARMM36m simulations. TIP3P (Transferable Intermolecular Potential with Three Points) water model^66^ was used in all simulations. The concentrations of KCl and NaCl were set to 150 mM.

After the CNGA1 channel was embedded into a POPC lipid bilayer with ions and water in a box, the simulation system was energy minimized and equilibrated. The energy minimization was done with GROMACS ‘steepest descent’ algorithm to reduce the system maximum force to below 1000 kJ/mol/nm in 50,000 steps. After the energy minimization, a 10 ns equilibration without any restraints was performed. For the simulations prepared by the CHARMM-GUI, the system was energy minimized and equilibrated in six steps using default scripts provided by the CHARMM-GUI.

For all simulation of the CNGA1 pore domain using CHARMM36m force field and all simulations of CNGA1 pore domain together with VSD and the whole channel construct, the transmembrane electric potential was directly generated by an external electric field applied along the z-axis (pore axis) (Supplementary Table S1). The voltage was calculated with:

1 $${{\rm{V}}}={{{\rm{EL}}}}_{{{\rm{z}}}}$$

where E denotes the applied electric field and L_z_ is the length of the simulation box along the z-axis^31,67,68^. The first 100 ns of these simulations (50 ns in closed structure simulations) were considered as equilibration phases and discarded for the further analysis (Supplementary Fig. S11).

For comparison purposes, for some simulations of the CNGA1 pore domain using AMBER force field, we used computational electrophysiology (CompEL) setup (Supplementary Table S1)^69^. To establish a transmembrane potential, a copy of the equilibrated system was stacked along the pore axis, generating two compartments (an inner and an outer). After building the two-bilayers system, we conducted another round of equilibration for 20 ns without cation imbalance before the actual MD production run. For the production run, a charge imbalance of 2 *e* between the two compartments separated by two lipid bilayers resulted in transmembrane voltages that are listed in Supplementary Table S1. During the MD simulations, ions passing through the pore were regularly monitored, and the charge imbalance was maintained by exchanging the same species of ion in one compartment with a water molecule from the other compartment^69^. The resulting transmembrane voltage can be calculated by double integration of the charge distribution using the Poisson equation as implemented in the GROMACS tool *gmx potential*^70^.

All atomistic molecular dynamics simulations were run on GROMACS software package (version 2021.1 and 2023.3)^71^. Short-range electrostatic interactions were calculated with a cutoff of 1.0 nm, whereas the long-range electrostatic interactions were treated using the particle mesh Ewald method^72^. The cutoff for van der Waals interaction was set to 1.0 nm. The simulations were performed at 300 K with an enhanced Berendsen thermostat (GROMACS V-rescale thermostat)^73^. The Parrinello- Rahman barostat^74^ was employed to keep the pressure within the system remaining at 1 bar. All bonds were constrained with the Linear Constraint Solver (LINCS) algorithm^75^. We used virtual sites approach to reduce the computational cost when adopting Amber99SB force field, which allowed us to increase the integration time step to 4 fs. Otherwise, all simulations were performed with an integration time step of 2 fs.

The protonation states of all titratable residues were assigned according to the standard protonation states at pH 7. Systems with varied protonation states at E365 were prepared by using CHARMM-GUI webserver tool^65^.

To investigate the influence of gate distance on the conductance, we applied harmonic distance restraints between the Cα atoms of the gate residue F389. We used the distances determined from the Cryo-EM structure and applied the distance restraints to both the opposite and adjacent subunits. Two distance restraints were applied on the opposite Cα of F389 with 1.93 nm and four distance restraints were applied on the adjacent Cα of F389 with 1.38 nm. The distance restraint force constant for all restraints was set to 1000 kJ/mol/nm^2^.

We calculated the pore radius profiles along the central axis of cryo-EM structure and during simulations of different constructs using HOLE program version 2.0^52,53^. Pore radius profiles during the simulations were computed from 10,000 frames, corresponding to 2000 frames extracted from a representative trajectory out of five independent MD simulation replicates.

All simulation details were summarized in Supplementary Table S1 and S2 and the root-mean-square-deviation (RMSD) analysis of the MD simulations was summarized in Supplementary Fig. S11.

### Trajectory analysis

All trajectories were analyzed with GROMACS toolkits and Python3 using MDAnalysis^76^ together with the package Numpy^77^, Matplotlib^78^, Pandas^79^ and SciPy^80^. Residue-residue pair distances were calculated using GROMACS tool *gmx dist* and dihedral angles were calculated with *gmx rama*. Presented data of dihedral angle distribution and residue-residue pair distance distribution were calculated using kernel density estimation^81^ from five parallel simulation runs. Ion permeation events were calculated when a cation permeated through the entire pore from the SF to the gate. To determine the ion hydration states during permeation, we calculated the number of ion-coordinating oxygens from both water molecules and protein residues within each ion’s first solvation shell. Hydration shells are dynamic; here, we defined the waters of hydration using the radii corresponding to the minimum in the radius of gyration profiles: 3.1 Å for Na^+^ and 3.4 Å for K^+^^82^. Instantaneous conductance was calculated by counting ion permeation events over 20 ns from five independent simulations. An ion permeation event was counted if the ion passed through the gate. Molecular visualizations were made with PyMol and Visual Molecular Dynamics (VMD)^83^.

### Statistics and reproducibility

Exact numbers of measurements are always provided in the respective figure descriptions. A two-tailed unpaired Student’s *t* test was performed and *p* values were calculated to assess the significance [not significant (ns): *p* > 0.05; significant (*): *p* ≤ 0.05; very significant (**): *p* ≤ 0.01; highly significant (***): *p* ≤ 0.001]. All the error bars represent the standard deviation (SD) of the mean.

### Reporting summary

Further information on research design is available in the Nature Portfolio Reporting Summary linked to this article.

## Supplementary information

Supplementary Information

Reporting Summary

## Data Availability

All data that support the findings of this study are included in this manuscript and in the Supplementary Information. All source data, Supplementary Movies and the simulation run input files, comprising the starting configuration and all necessary parameters for performing the MD simulations are deposited in Zenodo under accession code 10.5281/zenodo.14508687.

## Code availability

Molecular dynamics simulation data were generated using GROMACS 2021.1/2023.3. All trajectories were analyzed with GROMACS tools and Python using MDAnalysis together with Numpy, Matplotlib, Pandas, and SciPy.
