# Supplementary material for: Atomistic mechanism of non-selective cation permeation in cyclic nucleotide-gated CNGA1 ion channel by molecular dynamics simulations
Source: Commun Biol. 2025 Aug 23;8:1272. doi: 10.1038/s42003-025-08705-5 (PMC12375067; doi:10.1038/s42003-025-08705-5)
Supplement: Supplementary file 1 — Supplementary Information [file 42003_2025_8705_MOESM1_ESM.pdf]

Supplementary Information for

Atomistic Mechanism of Non-selective Cation Permeation in Cyclic Nucleotide-Gated CNGA1 Ion Channel by Molecular Dynamics Simulations

Haoran Liu[1,2], Johann Biedermann[1], Han Sun[1,2]*

[1]Research Unit of Structural Chemistry & Computational Biophysics, Leibniz-Forschungsinstitut für Molekulare Pharmakologie, Berlin 13125, Germany.

[2]Department of Chemistry, Technische Universität Berlin, Berlin 10623, Germany.

*corresponding author: hsun@fmp-berlin.de

Table of Content

Supplementary Figures
Supplementary Tables
Supplementary References

Supplementary Figures

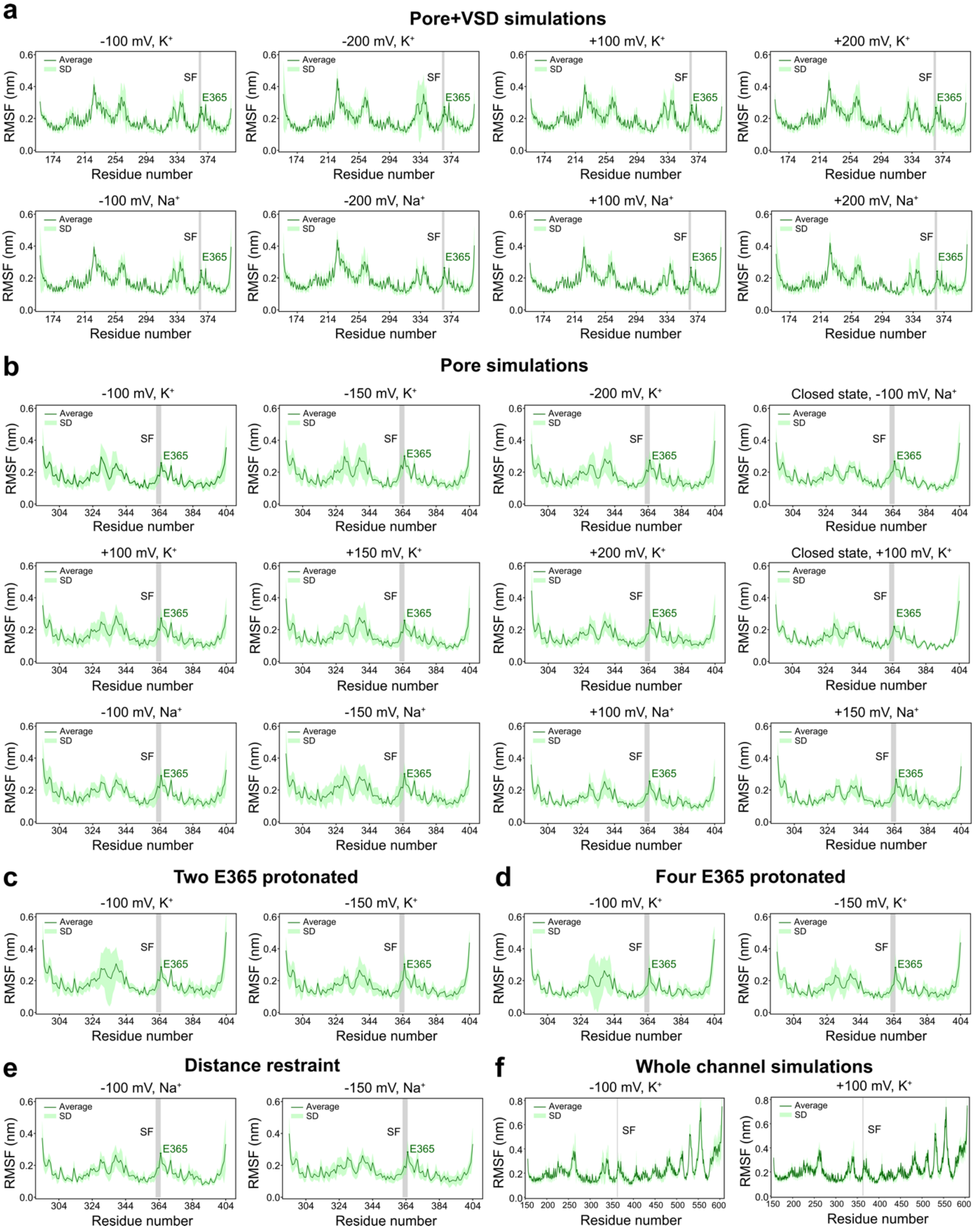

Supplementary Fig. S1 | Root-mean-square-fluctuation (RMSF) of the MD simulations. For each MD simulation setup, the green line represents the average RMSF value, while the light green shading indicates the standard deviation from five independent simulation runs. The grey-highlighted region marks the selectivity filter (SF), with E365 showing increased dynamics. **a** Simulations of the pore domain and the voltage-sensor domain (VSD) of the CNGA1 channel with K^+ and Na^+ under various transmembrane voltages. **b** Simulations of only the pore domain of the CNGA1 channel with K^+ and Na^+ under various transmembrane

voltages. **c** Simulations of the pore domain of the CNGA1 channel, where two opposing E365 residues are protonated, with K^+ under -100 mV and -150 mV voltages. **d** Simulations of the pore domain of the CNGA1 channel, where all four E365 residues are protonated, with K^+ under -100 mV and -150 mV voltages. **e** Simulations of the pore domain of the CNGA1 channel with distance restraints on gate residue F389 with Na^+ under -100 mV and -150 mV voltages. **f** Simulations of the whole CNGA1 channel with K^+ under -100 mV and +100 mV voltages.

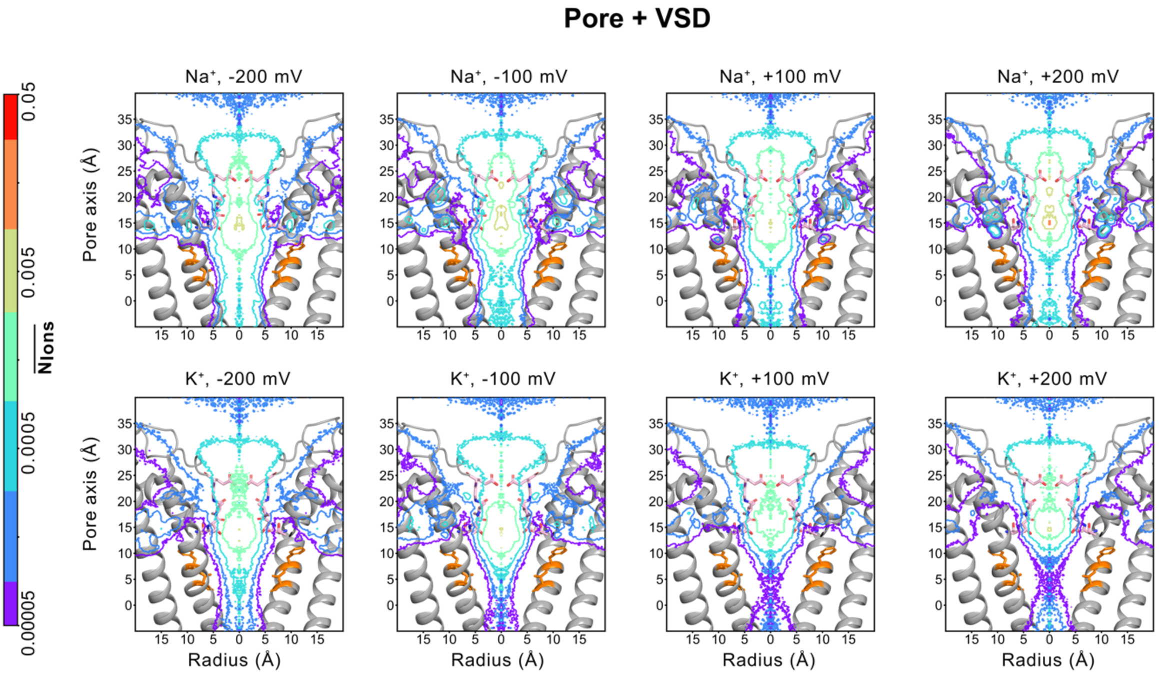

Supplementary Fig. S2 | Ion binding profiles of K^+ and Na^+ within the ion conduction pathway derived from MD simulations of the pore domain together with the voltage-sensor domain (VSD). Comparison of 2-dimensional ion occupancy for the CNGA1 channel under varying voltage conditions with Na^+ and K^+. The ion occupancy in number of ions per 0.001 Å^3 per 50 ps was normalized according to the volume change along the radius. The simulations were performed with the pore domain together with the VSD.

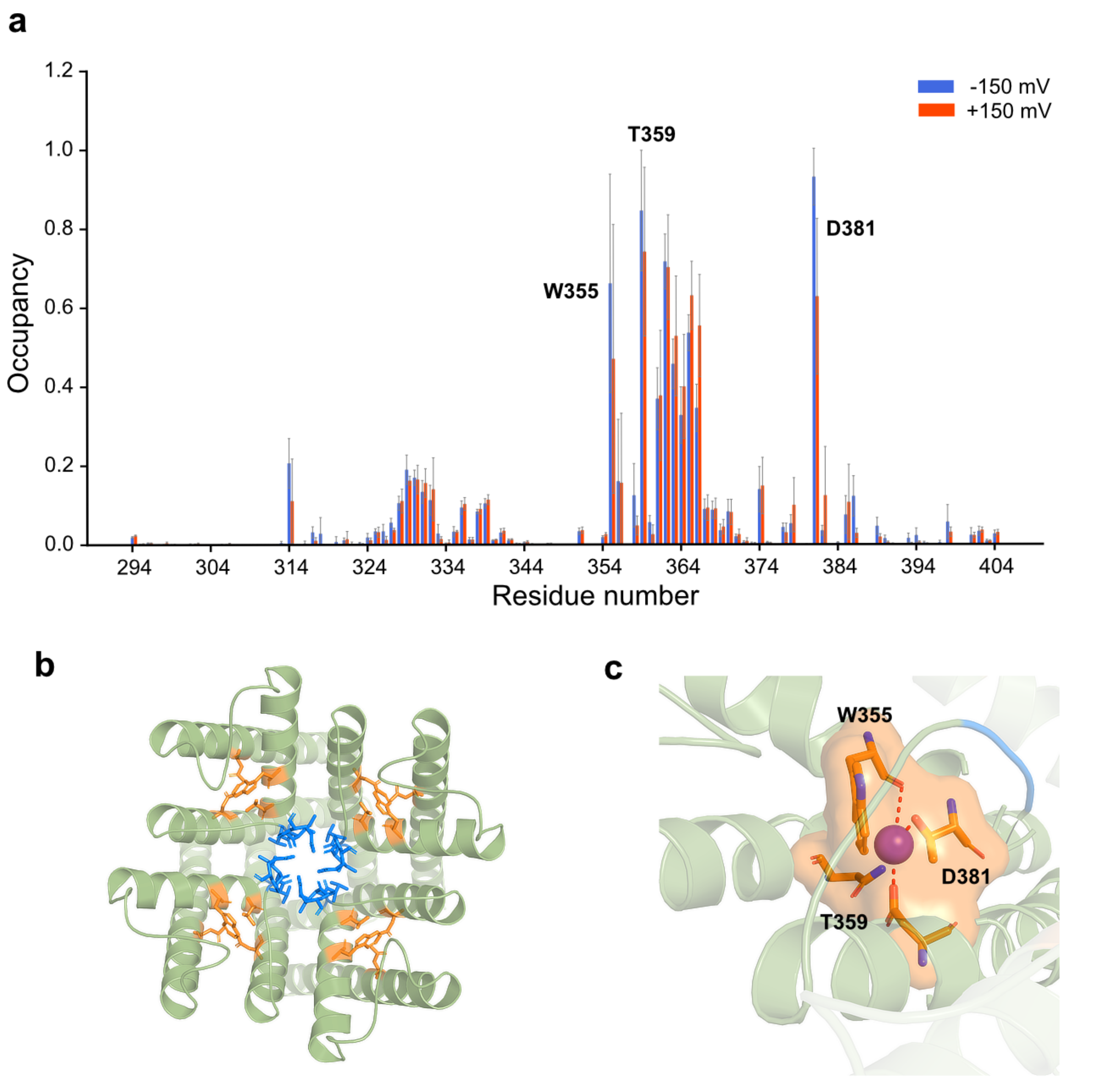

Supplementary Fig. S3 | Off-axis binding site behind the SF. **a** Residue-wise K^+ occupancy of pore-domain only simulations of the CNGA1 under -150 mV (blue) and +150 mV (red) respectively. The occupancy was calculated from five independent simulation runs of 1000 ns of each. All the error bars represent the standard deviation of the mean. **b, c** off-axis binding site behind the SF, consisting of the backbone of W355 and side-chains of T359 and D381.

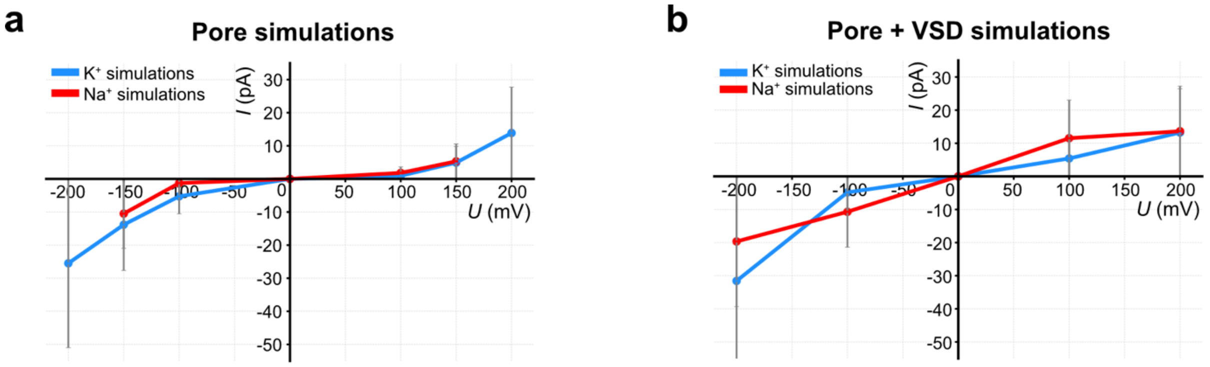

Supplementary Fig. S4 | The simulated current-voltage relationship for different constructs of the CNGA1 channel. a The current-voltage relationship for the pore only simulations with K^+ (blue) and Na^+ (red). **b** The current-voltage relationship for the pore+VSD simulations with K^+ (blue) and Na^+ (red). The standard deviations derived from five simulation replicas are shown as gray lines.

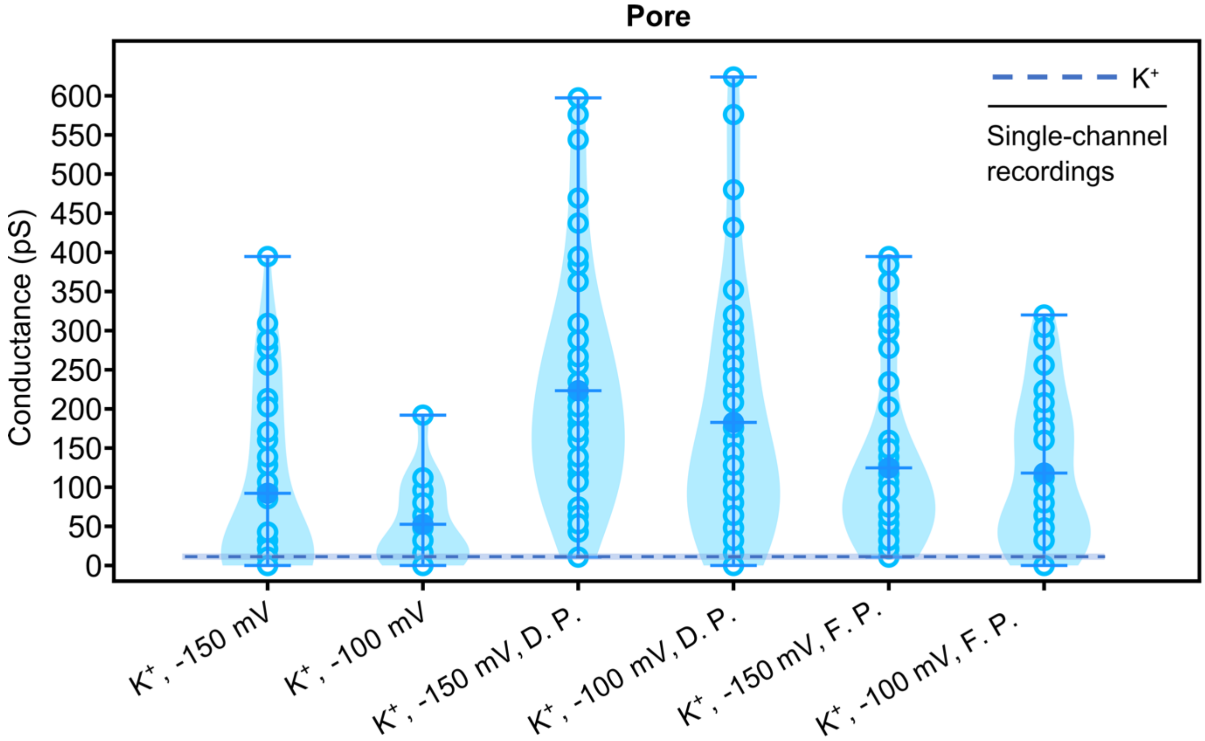

Supplementary Fig. S5 | Comparison of conductance across different protonation states of E365. The first two rows represent the simulations with all E365 residues of four subunits in their deprotonated state. ''D. P.'' represent the simulations with two diagonal E365 residues protonated. ''F. P.'' represent the simulations with four E365 residues protonated. Open circle represents the simulated conductance from each 100 ns segment, in total 45 data points for each simulation setup, while the closed circle shows the averaged value from all data points.

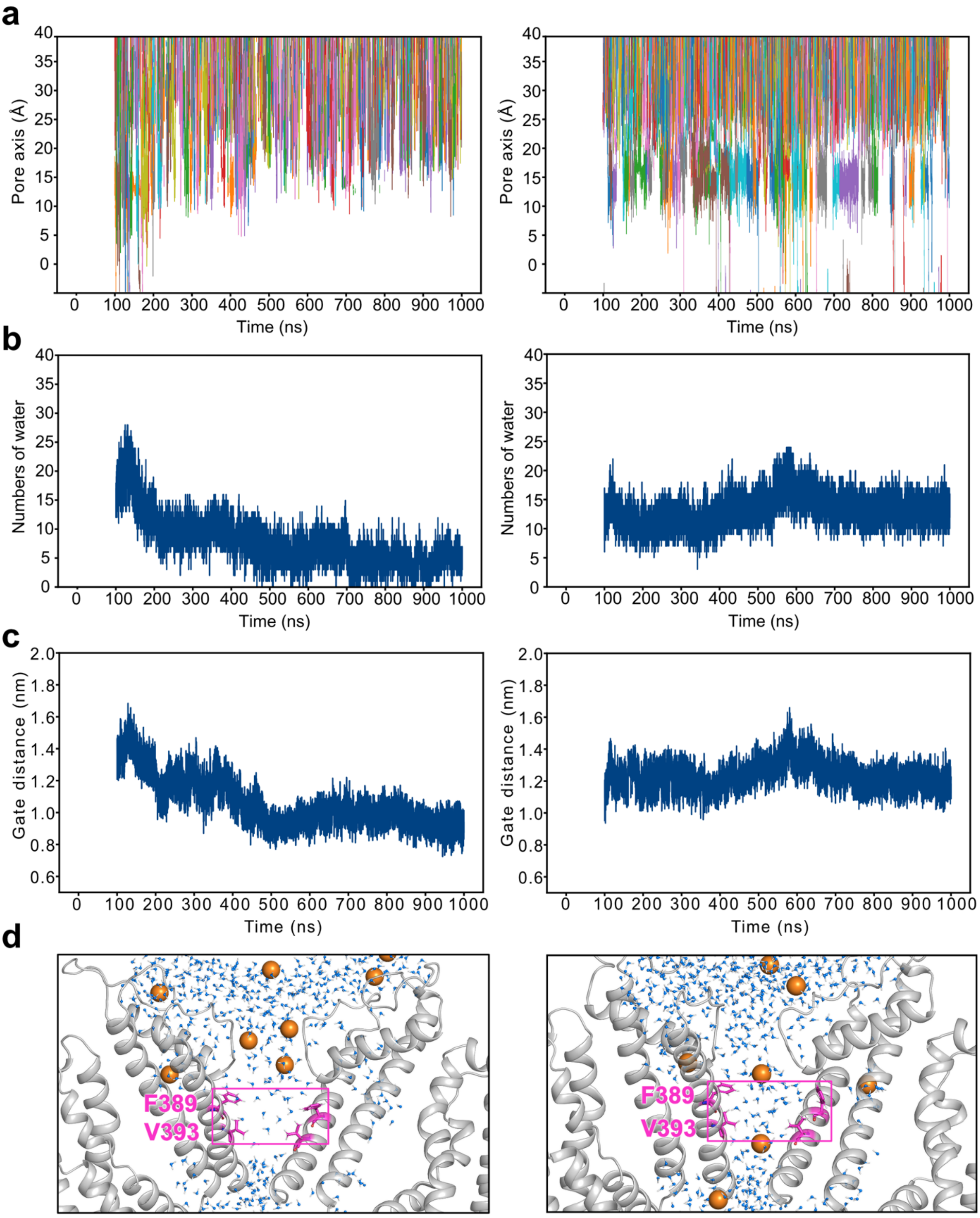

Supplementary Fig. S6 | Examples illustrating ion permeations arrest due to partial gate tightening and dehydration. a Representative traces of K^+ ions passing through the pore of the CNGA1 channel. The left panel shows a trajectory where ion flow stops after 100 ns, while the right panel shows continuous ion permeations throughout the simulations. **b** Time course of the number of water molecules in the gate region during the simulations shown in (**a**). **c** Distance between the opposing gate residues F389 over time in the simulations shown in (**a**). **d** Two representative snapshots illustrating differences in hydration levels in the gate region: the left panel shows partial dehydration, while the right panel shows full hydration.

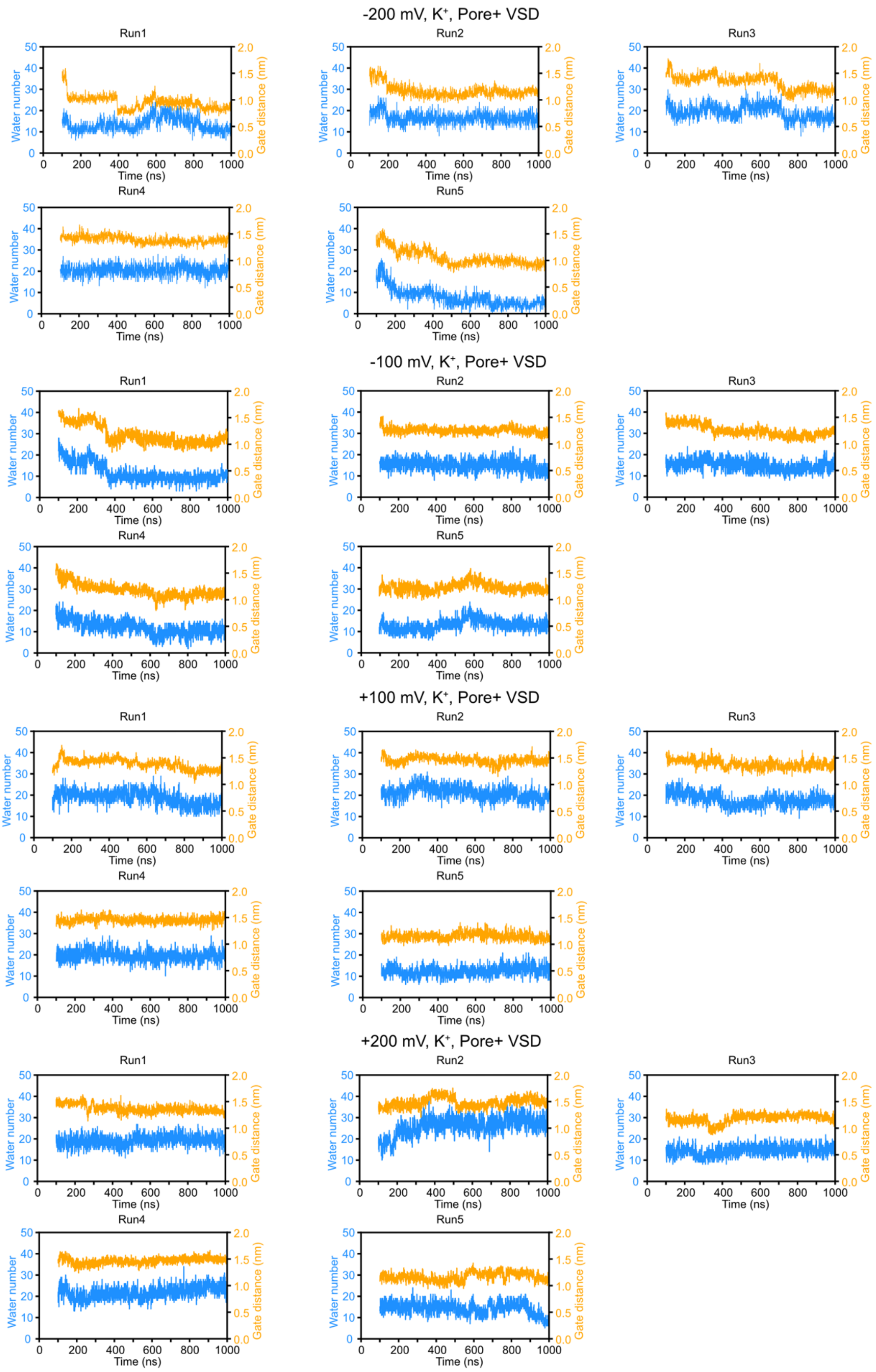

Supplementary Fig. S7 | The number of water molecules in the gate region (blue) and the average distance between the two opposing gate residues (F389, yellow) during the simulations with K^+. The simulations were performed with the pore domain together with the VSD.

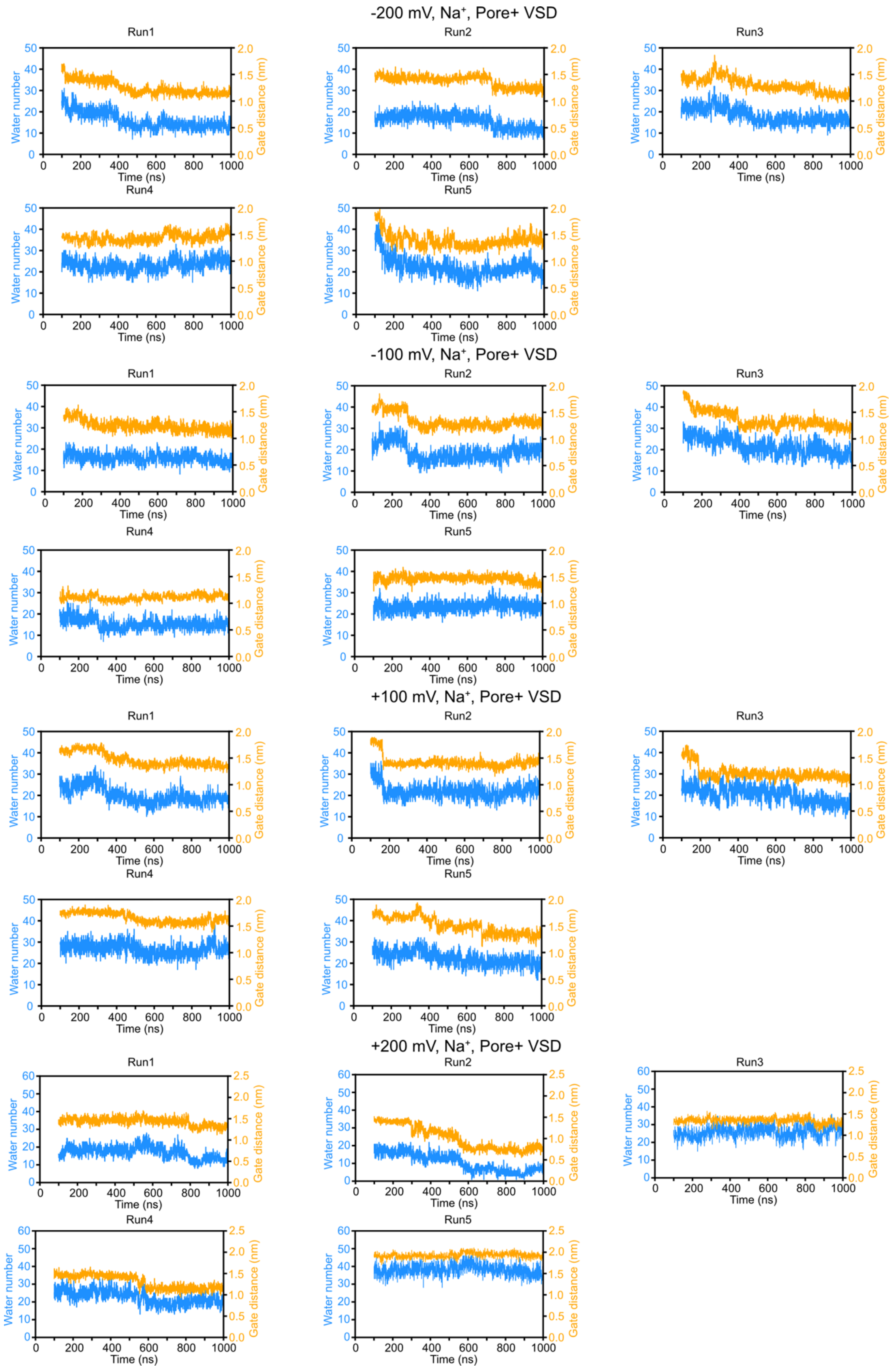

Supplementary Fig. S8 | The number of water molecules in the gate region (blue) and the average distance between the two opposing gate residues (F389, yellow) during the simulations with Na^+. The simulations were performed with the pore domain together with the VSD.

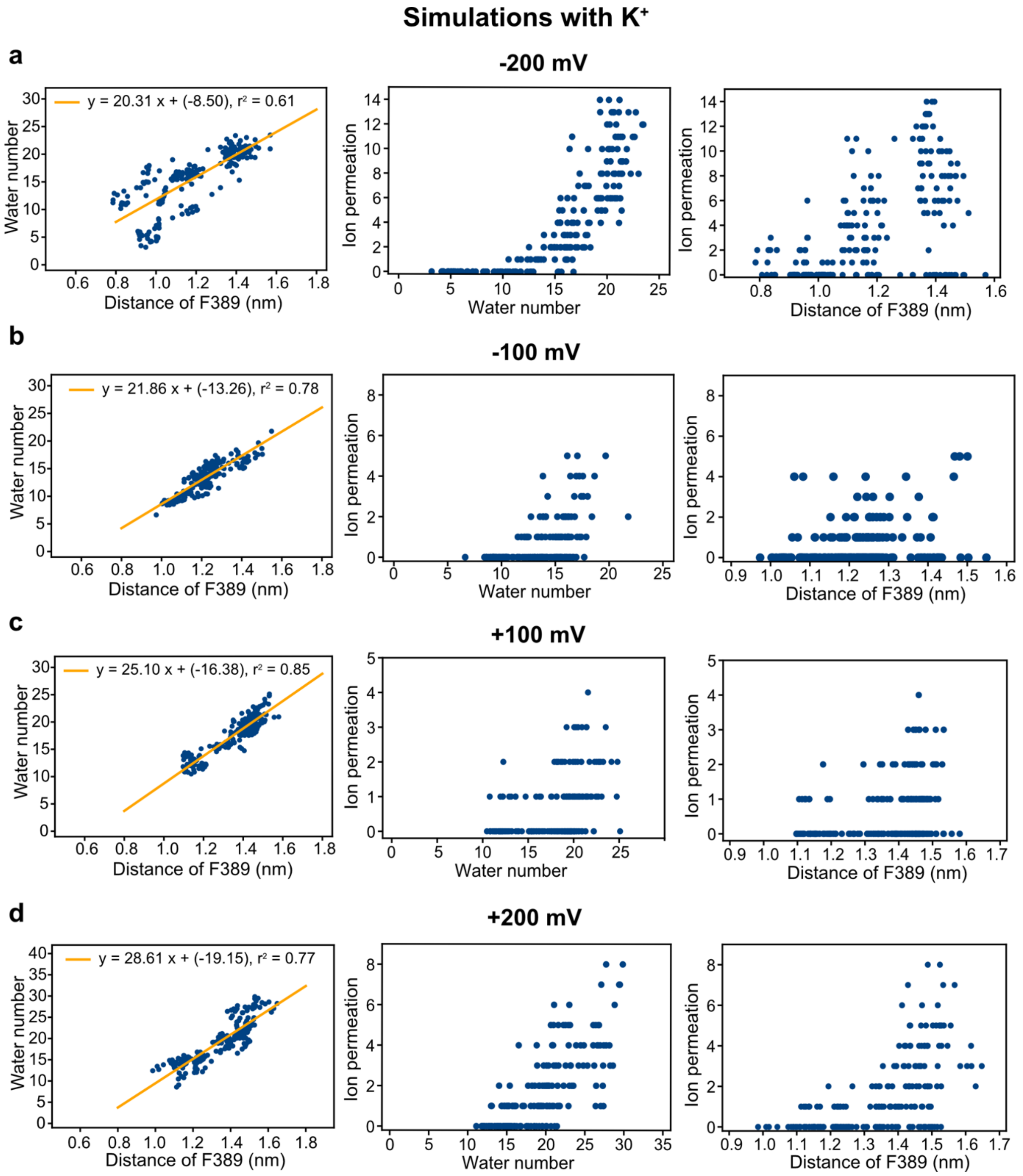

Supplementary Fig. S9 | The relationship between the average number of water molecules in the gate region per 20 ns interval, the average distance between the two opposing gate residues (F389), and the number of ion permeation events during the same time interval. K^+ permeation simulations were performed at -200 mV, -100 mV, +100 mV, and +200 mV, respectively. The simulations were performed with the pore domain together with the VSD.

Simulations with Na^+

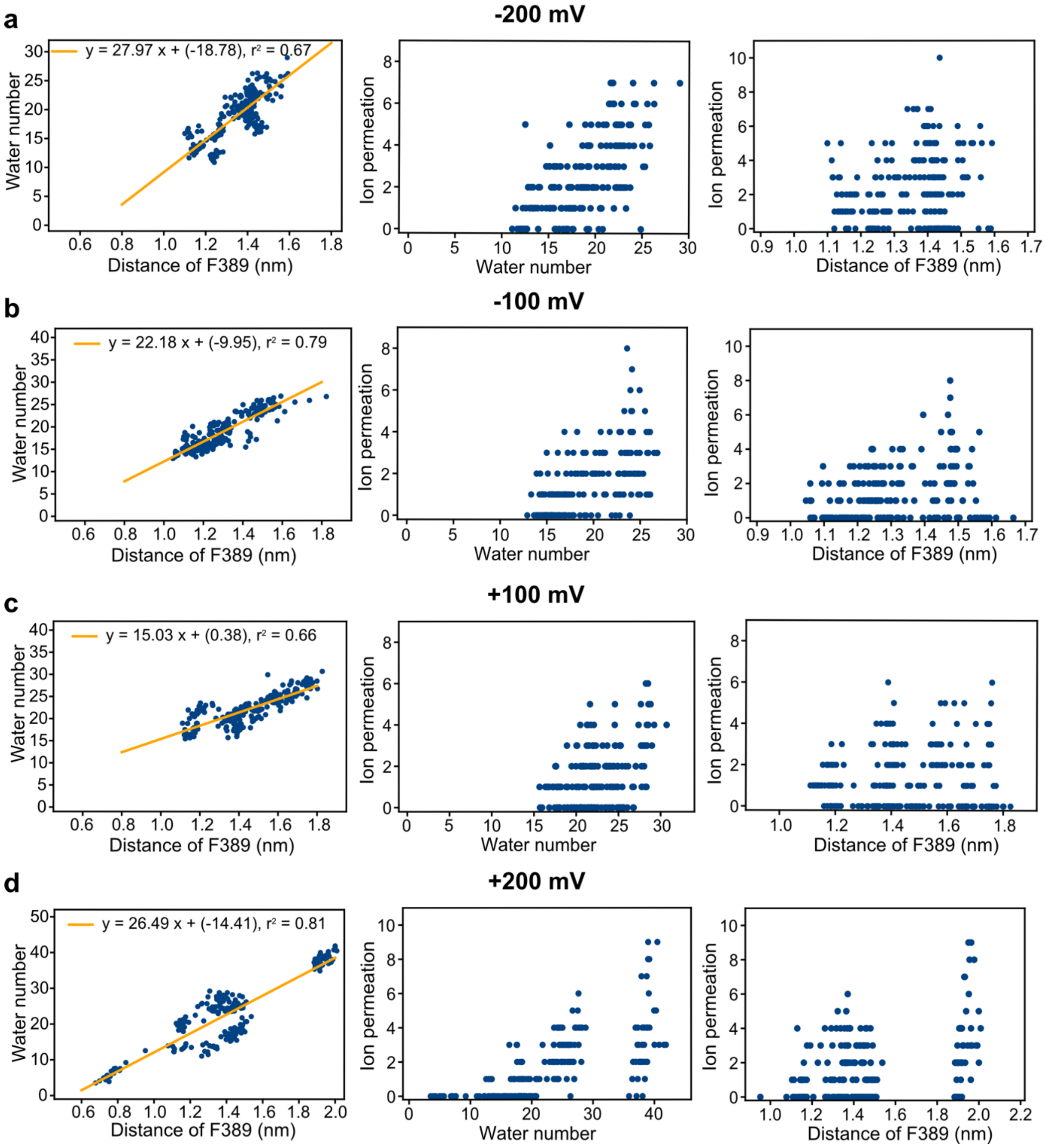

Supplementary Fig. S10 | The relationship between the average number of water molecules in the gate region per 20 ns interval, the average distance between the two opposing gate residues (F389), and the number of ion permeation events during the same time interval. Na^+ permeation simulations were performed at -200 mV, -100 mV, +100 mV, and +200 mV, respectively. The simulations were performed with the pore domain together with the VSD.

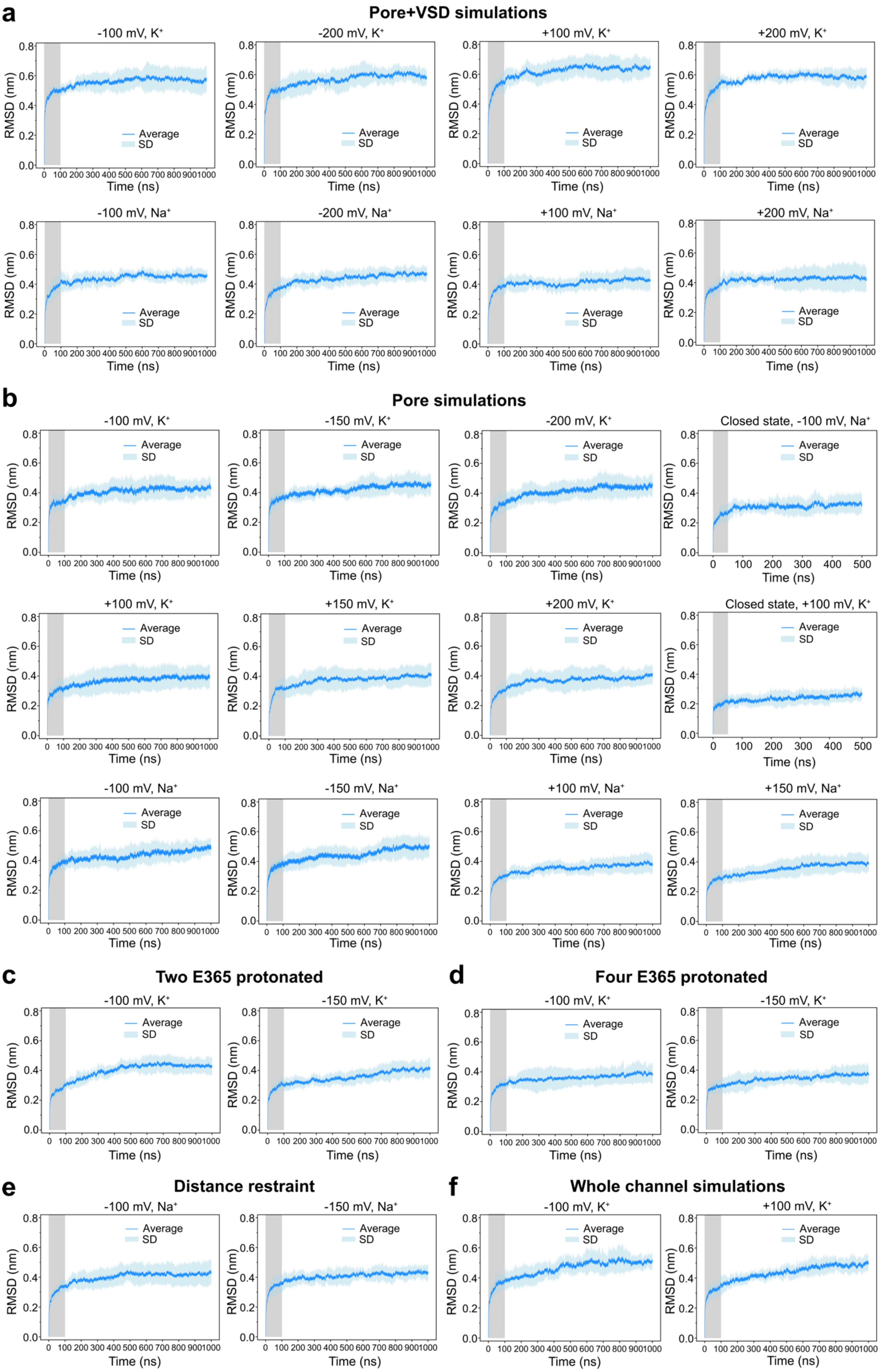

a
Pore+VSD simulations
-100 mV, K⁺
-200 mV, K⁺
+100 mV, K⁺
+200 mV, K⁺
-100 mV, Na⁺
-200 mV, Na⁺
+100 mV, Na⁺
+200 mV, Na⁺
b
Pore simulations
-100 mV, K⁺
-150 mV, K⁺
-200 mV, K⁺
Closed state, -100 mV, Na⁺
+100 mV, K⁺
+150 mV, K⁺
+200 mV, K⁺
Closed state, +100 mV, K⁺
-100 mV, Na⁺
-150 mV, Na⁺
+100 mV, Na⁺
+150 mV, Na⁺
c
Two E365 protonated
-100 mV, K⁺
-150 mV, K⁺
d
Four E365 protonated
-100 mV, K⁺
-150 mV, K⁺
e
Distance restraint
-100 mV, Na⁺
-150 mV, Na⁺
f
Whole channel simulations
-100 mV, K⁺
+100 mV, K⁺
RMSD (nm)
Time (ns)
Average
SD
0.0
0.2
0.4
0.6
0.8
0 100 200 300 400 500 600 700 800 900 1000
0 100 200 300 400 500

Supplementary Fig. S11 | Root-mean-square-deviation (RMSD) of the MD simulations. For each MD simulation setup, the blue line represents the average RMSD value, with the blue shading indicating the standard deviation from five independent simulation runs. The grey-shaded area illustrates the equilibration period for each simulation: 100 ns for 1 μs and 50 ns for the 500 ns simulations. The equilibration periods were excluded from the subsequent analyses. **a** Simulations involving the pore domain and voltage-sensor domain (VSD) of the CNGA1 channel with K^+ and Na^+ under various transmembrane voltages. **b** Simulations involving only the pore domain of the CNGA1 channel with K^+ and Na^+ under various transmembrane voltages. **c** Simulations of the pore domain of the CNGA1 channel, where two opposing E365 residues are protonated, with K^+ under -100 mV and -150 mV voltages. **d** Simulations of the pore domain of the CNGA1 channel, where all four E365 residues are protonated, with K^+ under -100 mV and -150 mV voltages. **e** Simulations of the pore domain of the CNGA1 channel with distance restraints on gate residues F389 with Na^+ under -100 mV and -150 mV voltages. **f** Simulations of the whole CNGA1 channel with K^+ under -100 mV and +100 mV voltages.

Supplementary Tables

Supplementary Table S1 | List of simulation systems. Simulations involving the CNGA1 channel pore domain or the pore domain together with the VSD for Na^+ and K^+ under different voltages. All of the simulations were performed with a salt concentration of 150 mM and a temperature at 300 K. * Simulations using computational electrophysiology method[1]; others using an external electric field[2].

Simulation system	Force field	Cation	Time (ns)	V (mV)	Replicates	Mean ion permeations	γ (pS)
Pore, closed	CHARMM36m	K^+	450	+100	5	0 ± 0	0 ± 0
Pore, closed	CHARMM36m	Na^+	450	-100	5	0 ± 0	0 ± 0
Pore	CHARMM36m	K^+	900	-100	5	25 ± 17	44 ± 31
Pore	CHARMM36m	K^+	900	-150	8	72 ± 66	86 ± 79
Pore	CHARMM36m	K^+	900	-200	5	122 ± 132	109 ± 118
Pore	CHARMM36m	K^+	900	+100	5	5 ± 4	10 ± 8
Pore	CHARMM36m	K^+	900	+150	5	36 ± 21	43 ± 25
Pore	CHARMM36m	K^+	900	+200	5	64 ± 21	57 ± 18
Pore	CHARMM36m	Na^+	900	-100	5	6 ± 9	11 ± 15
Pore	CHARMM36m	Na^+	900	-150	8	76 ± 67	90 ± 80
Pore	CHARMM36m	Na^+	900	+100	5	10 ± 8	19 ± 14
Pore	CHARMM36m	Na^+	900	+150	5	29 ± 17	34 ± 20
Pore, two E365 protonated	CHARMM36m	K^+	900	-100	5	92 ± 77	164 ± 138
Pore, two E365 protonated	CHARMM36m	K^+	900	-150	5	179 ± 62	212 ± 74
Pore, four E365 protonated	CHARMM36m	K^+	900	-100	5	65 ± 41	116 ± 74
Pore, four E365 protonated	CHARMM36m	K^+	900	-150	5	101 ± 76	119 ± 90
Pore, distance restraint	CHARMM36m	Na^+	900	-100	5	15 ± 15	27 ± 27
Pore, distance restraint	CHARMM36m	Na^+	900	-150	5	25 ± 17	29 ± 20
Pore + VSD	CHARMM36m	K^+	900	-100	5	24 ± 9	43 ± 17
Pore + VSD	CHARMM36m	K^+	900	-200	8	165 ± 126	146 ± 111
Pore + VSD	CHARMM36m	K^+	900	+100	5	24 ± 13	43 ± 23
Pore + VSD	CHARMM36m	K^+	900	+200	5	54 ± 49	48 ± 43
Pore + VSD	CHARMM36m	Na^+	900	-100	5	60 ± 40	107 ± 71
Pore + VSD	CHARMM36m	Na^+	900	-200	5	110 ± 41	98 ± 36
Pore + VSD	CHARMM36m	Na^+	900	+100	5	64 ± 43	114 ± 78
Pore + VSD	CHARMM36m	Na^+	900	+200	5	76 ± 54	68 ± 48
Pore *	AMBER99SB	K^+	500	+194 ± 79	5	13 ± 3	21 ± 6
Pore *	AMBER99SB	K^+	500	-194 ± 79	5	49 ± 23	80 ± 38
Pore *	AMBER99SB	Na^+	500	+145 ± 89	5	1 ± 1	2 ± 2
Pore *	AMBER99SB	Na^+	500	-145 ± 89	5	1 ± 1	2 ± 2
Pore *	AMBER19SB	K^+	500	+154 ± 67	5	8 ± 2	17 ± 5
Pore *	AMBER19SB	K^+	500	-154 ± 67	5	59 ± 36	123 ± 75
Pore *	AMBER19SB	Na^+	500	+111 ± 56	5	6 ± 3	18 ± 11
Pore *	AMBER19SB	Na^+	500	-111 ± 56	5	1 ± 1	4 ± 3
Whole channel	CHARMM36m	K^+	900	+100	5	41 ± 13	73 ± 23
Whole channel	CHARMM36m	K^+	900	-100	5	76 ± 52	135 ± 92

Supplementary Table S2 | Simulation system details. The simulations without special indication were performed with the CHARMM36m force field. * Simulations using computational electrophysiology method[1]; others using an external electric field[2].

Simulation system	Cation	Number of protein atoms	Number of water molecules	Number of POPC	Number of cations	Number of Cl^-	System atoms in total
Pore, closed	K^+	7188	23752	237	66	58	110326
Pore, closed	Na^+	7188	23748	237	66	58	110314
Pore	K^+	7188	23590	235	66	58	109572
Pore	Na^+	7188	23594	235	66	58	109584
Pore, two E365 protonated	K^+	7170	23595	235	68	58	109571
Pore, four E365 protonated	K^+	7172	23589	235	66	58	109553
Pore, distance restraint	Na^+	7188	23594	235	66	58	109584
Pore + VSD	K^+	16652	25740	246	74	66	126976
Pore + VSD	Na^+	16632	25960	236	77	65	126278
Pore, AMBER19SB *	K^+	14376	47174	470	132	116	219126
Pore, AMBER19SB *	Na^+	14376	47164	470	132	116	219096
Pore, AMBER99SB *	K^+	15752	45666	590	140	124	183694
Pore, AMBER99SB *	Na^+	15752	45666	590	140	124	183694
Whole channel	K^+	29760	42563	319	109	109	200413

Supplementary Table S3 | Number of ions entering the SF via off-axis pathway and on-axis pathway. All simulations were performed with the pore-domain only simulations of the CNGA1 using the CHARMM36m force field.

Voltages	off-axis pathway	On-axis pathway	Percentage of off-axis pathway
-150 mV	108	8225	1.3%
-100 mV	48	6236	0.8%
+100 mV	2	24	7.7%
+150 mV	0	114	0%

Supplementary References

1. Kutzner C, Grubmuller H, de Groot BL, Zachariae U. Computational Electrophysiology: The Molecular Dynamics of Ion Channel Permeation and Selectivity in Atomistic Detail. *Biophysical Journal* **101**, 809-817 (2011).

2. Caleman C, van der Spoel D. Picosecond melting of ice by an infrared laser pulse: a simulation study. *Angew Chem Int Ed Engl* **47**, 1417-1420 (2008).
